# Theoretical and Anti-*Klebsiella pneumoniae* Evaluations of Substituted 2,7-dimethylimidazo[1,2-a]pyridine-3-carboxamide and Imidazopyridine Hydrazide Derivatives

**DOI:** 10.3390/molecules28062801

**Published:** 2023-03-20

**Authors:** Suraj N. Mali, Amit Anand, Magdi E. A. Zaki, Sami A. Al-Hussain, Rahul D. Jawarkar, Anima Pandey, Aleksey Kuznetsov

**Affiliations:** 1 Department of Pharmaceutical Sciences and Technology, Birla Institute of Technology, Ranchi 835215, India; 2 Department of Chemistry, Faculty of Science, Imam Mohammad Ibn Saud Islamic University (IMSIU), Riyadh 11623, Saudi Arabia; 3 Department of Medicinal Chemistry and Drug Discovery, Dr. Rajendra Gode Institute of Pharmacy, University Mardi Road, Amravati 444603, India; 4 Department of Chemistry, Universidad Técnica Federico Santa Maria, Santiago 7660251, Chile

**Keywords:** theoretical study, in vitro antibacterial activity, DFT, TD-DFT, imidazopyridines

## Abstract

A series of multistep synthesis protocols was adopted to synthesize substituted imidazopyridines (IMPs) (SM-IMP-01 to SM-IMP-13, and DA-01-05). All substituted IMPs were then characterized using standard spectroscopic techniques such as ^1^H-NMR, ^13^C-NMR, elemental analyses, and mass spectrometry. Our both in vitro qualitative and quantitative results for antibacterial analysis, against *Klebsiella pneumoniae ATCC 4352* and *Bacillus subtilis ATCC 6051* suggested that all compounds essentially exhibited activity against selected strains of bacteria. Our DFT analyses suggested that the compounds of the SM-IMP-01–SM-IMP-13 series have HOMO/LUMO gaps within 4.43–4.69 eV, whereas the compounds of the DA-01–DA-05 series have smaller values of the HOMO/LUMO gaps, 3.24–4.17 eV. The lowest value of the global hardness and the highest value of the global softness, 2.215 and 0.226 eV, respectively, characterize the compound SM-IMP-02; thus, it is the most reactive compound in the imidazopyridine carboxamide series (except hydrazide series). This compound also depicted lesser MIC values against *Klebsiella pneumoniae ATCC 4352* and *Bacillus subtilis ATCC 6051* as 4.8 µg/mL, each. In terms of another series, hydrazide DA-05 depicted strong antimicrobial actions (MIC: 4.8 µg/mL against both bacterial strains) and also had the lowest energy gap (3.24 eV), higher softness (0.309 eV), and lesser hardness (1.62 eV). Overall, when we compare qualitative and quantitative antimicrobial results, it is been very clear that compounds with dibromo substitutions on imidazopyridine (IMP) rings would act as better antimicrobial agents than those with -H at the eighth position on the IMP ring. Furthermore, substituents of higher electronegativities would tend to enhance the biological activities of dibromo-IMP compounds. DFT properties were also well comparable to this trend and overall, we can say that the electronic behavior of compounds under investigation has key roles in their bioactivities.

## 1. Introduction

Antimicrobial resistance (AMR) endangers the ability to prevent and treat a growing variety of infections caused by bacteria, parasites, viruses, and fungi [1,2]. AMR is the consequence of changes that occur in bacteria, viruses, fungi, and parasites over time, rendering them unresponsive to medications [3,4]. This can result in challenges in managing infections, increased risks of disease transmission, severe illness, and even fatalities [3]. It also leads to medication inefficacy and prolonged infection in the body, elevating the risk of spreading the disease to others. This issue points out the fact that there is an unmet need to develop safer, and more potent antimicrobial agents [5,6,7,8,9,10,11,12] with unique mechanisms of action.

Heterocyclic structures containing nitrogen-based rings are part of many marketed drugs. Imidazopyridines and fused imidazopyridines rings are common examples of such rings and are found in a variety of bioactive compounds [7,11]. These rings have unique properties, such as high polarity and the ability to participate in hydrogen bonding and coordination chemistry, which allow them to interact with different biomolecules. As a result, compounds containing imidazopyridines rings have a diverse range of biological activities. Imidazopyridine-based compounds have been widely reported for a number of biological activities, including but not limited to antimicrobial, antibacterial, anticancer, and antiviral activities [13,14,15,16,17].

Hydrazide-hydrazones belong to the carbonyl category and contain an azomethine group (-NH-H=CH-) [8,10]. In the last two decades, they have become a popular moiety in research due to their significant role as intermediates [8]. Researchers find this topic very intriguing because of the broad range of promising biological activities associated with this compound, such as anti-microbial, anti-cancer, anti-convulsant, anti-tubercular, anti-inflammatory, and analgesic activities [18,19,20,21,22,23,24,25]. The basic structure of hydrazones includes two nitrogen (-NNH_2_) and one carbon (C=O) atom, which combine to form a C=N bond through the conjugation of a lone pair of electrons from nitrogen [8].

In the present work, a multistep synthesis procedure was adopted to synthesize 13 previously known IMPCs (2,7-dimethylimidazo [1,2-a]pyridine-3-carboxamides) [26] along with synthesizing 5 new imidazopyridine-based hydrazides, too. All 18 synthesized compounds were then characterized using standard analytical techniques such as FTIR (Fourier transform infrared spectroscopy), NMR (nuclear magnetic resonance spectroscopy), mass, and elemental analyses and subjected to in vitro anti-bacterial analysis on selected strains (please refer to Figures S1–S60 for spectral data). Furthermore, the DFT (density functional theory) method is used to calculate the global chemical reactivity descriptors of the title molecules, such as chemical hardness, energy, electronic chemical potential, and electrophilicity [27,28,29,30,31,32,33,34,35,36,37,38,39,40,41]. These descriptors are employed to predict the relative stability and reactivity of the molecules.

## 2. Results

### 2.1. Structures and Energetics

**Figure 1:**
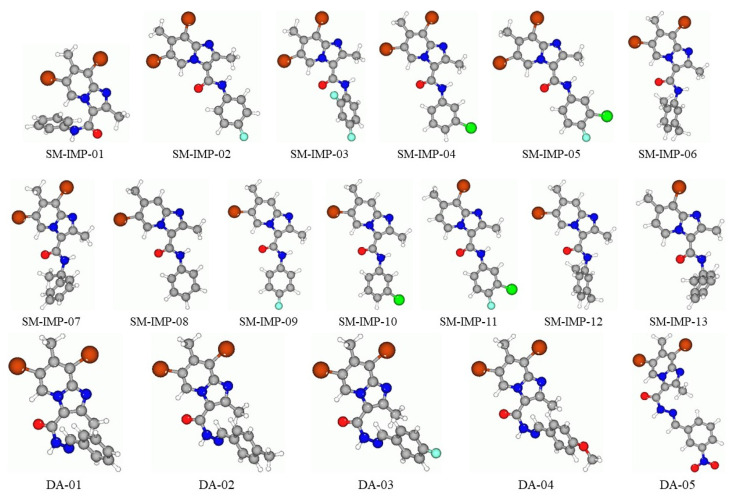
Optimized structures for the two series of the investigated compounds, B3LYP/6-311+G(d,p), with implicit effects from water.

In Figure 1 the structures of the two-compound series optimized with the implicit effects from water are shown and Table 1 summarizes the electronic energies, HOMO/LUMO energies, and the HOMO/LUMO and TDDFT gaps of these compounds. As can be seen from Figure 1, several compounds of the first series, SM-IMP-02, SM-IMP-04, SM-IMP-05, SM-IMP-08–SM-IMP-11 have essentially completely flat structures; whereas other compounds of this series have the structures distorted in some way due to rotations of phenyl rings. The compounds of the second series all have non-flat structures. These results imply that molecules of the compounds of both series could adjust themselves well to structures of proteins and other biopolymers thus allowing noticeable possible intermolecular interactions and resulting bioactivity.

The data in Table 1 show that the compounds of the SM-IMP-01–SM-IMP-13 series have HOMO/LUMO gaps within 4.43–4.69 eV, whereas the compounds of the DA-01–DA-05 series have smaller values of the HOMO/LUMO gaps, 3.24–4.17 eV. This would imply that the compounds of the second series should be more reactive and less stable than the compounds of the first series (see below discussion of the GRPs). According to Mabkhot et al. [40], the HOMO-LUMO energy gap (E gap) is an established parameter to measure the extent of the intramolecular charge transfer and was used in pharmaceutical studies. In this study, the compounds exhibited different antimicrobial activity, this may be due to the difference in HOMO-LUMO energy gap levels. The lower value of the HOMO and LUMO energy gap showed that the studied molecule has high chemical reactivity, and biological activity, herein, antimicrobial actions. Such a relationship can also be analogous with the earlier literature-reported explanations [4,7,11,41]. 

In the SM-IMP-01–SM-IMP-13 series, SM-IMP-02 and SM-IMP-05 have the smallest HOMO/LUMO gap values, 4.43 and 4.45 eV, respectively, whereas SM-IMP-13 has the largest HOMO/LUMO gap value, 4.69 eV. Therefore, we can suggest that the first two compounds would have the highest reactivity in the first series and the last compound would be the least reactive one. Further, in the DA-01–DA-05 series, DA-05 has the smallest value of the HOMO/LUMO gap, 3.24 eV, whereas DA-01 has the largest gap value, 4.17 eV, thus implying that DA-05 would be the most reactive and DA-01 would be the least reactive species.

### 2.2. NPA Charges

**Figure 2:**
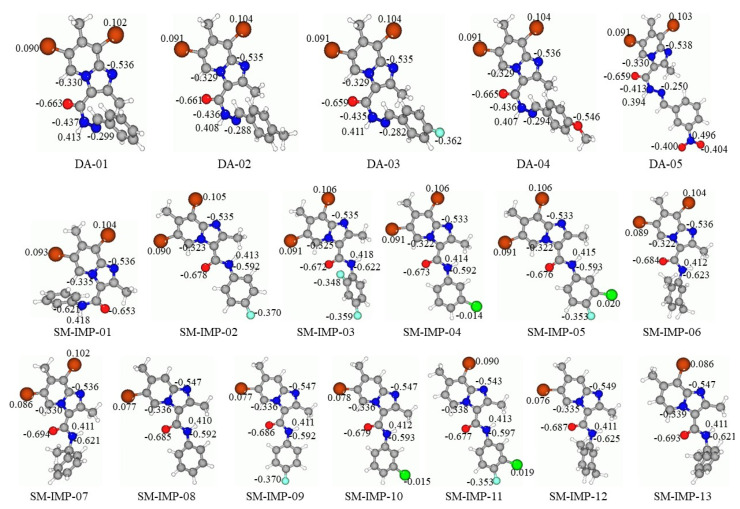
NPA charges, e, of selected atoms of the two series of the investigated compounds, B3LYP/6-311+G(d,p), computed with implicit effects from water.

The calculated NPA charges on selected atoms (heteroatoms) of the compounds of both series (Figure 2) imply that numerous electrostatic and dispersion interactions, as well as inter- and intramolecular hydrogen bonds, can be formed between molecules of these compounds and fragments of biomolecules with which they interact, e.g., amino acids. This suggests the formation of quite strong complexes of these molecules with various biomolecules and thus their potential as pharmacologically active compounds. It is worthwhile to note that charges on Br atoms in the series SM-IMP-01–SM-IMP-07 are very close to charges on Br atoms in the DA-01–DA-05 series. However, for the SM-IMP-08–SM-IMP-13 compounds with only one Br atom its charge depends on the position relative to the carbonyl oxygen. Charges on the carbonyl oxygen and amido group nitrogen in the compounds of the SM-IMP series would depend on the molecular conformation and the type of ligands bound to the amido group. In the DA compound series, charges on these atoms are significantly modified when the phenyl with a meta-nitro group is present in the molecule, like in the DA-05 compound. Thus, using different ligands in the compounds of both series would allow modifying charges on the amido group atoms, which, along with various substituents in the ligand (halogens, alkyls, nitro groups, etc.), might help to modify the binding of these compounds with their target molecules and thus influence their pharmacological activity.

### 2.3. Frontier MOs

**Figure 3:**
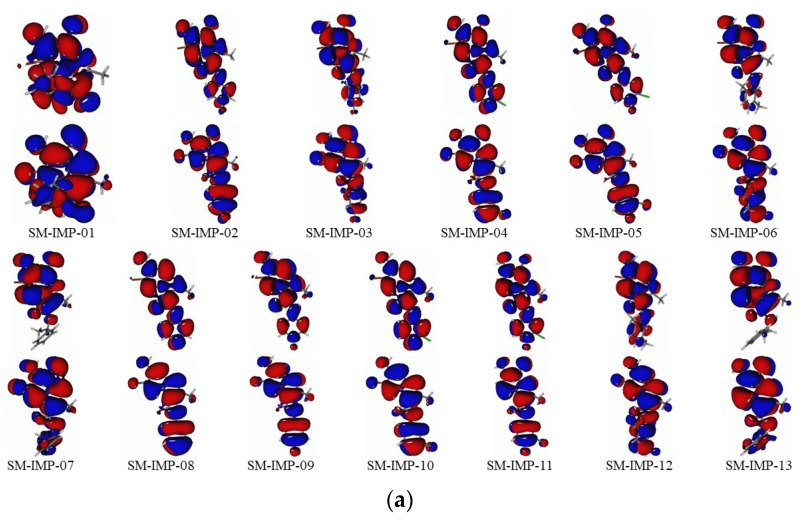

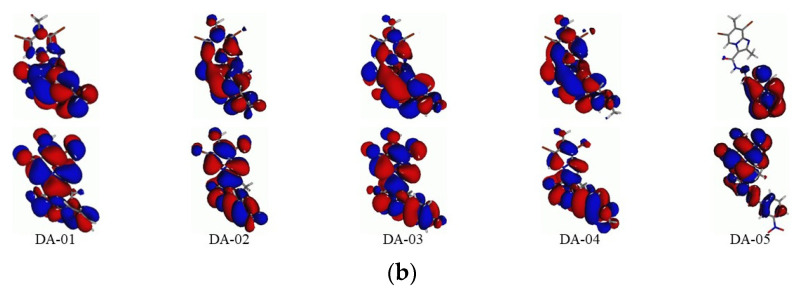
The contour plots of HOMOs and LUMOs for the series SM-IMP-01 to SM-IMP-13 (**a**) and DA-01 to DA-05 (**b**), B3LYP/6-311+G(d,p), implicit water. HOMOs are shown in the bottom row and LUMOs are shown in the top row for each compound.

Consideration of FMOs for both series provided in Figure 3 shows noticeable similarities of the HOMOs and LUMOs for almost all compounds: the FMOs of almost all compounds in both series are contributed by essentially whole molecules, with exception of the LUMOs of SM-IMP-07 and SM-IMP-13 (Figure 3a), which do not have contributions from the phenyls and alkyls of the ligands attached to the amide bonds, and the LUMO of DA-05, which is, on the contrary, dominated by contributions of the ligand containing the nitro group. These results imply that in binding interactions and chemical reactions with biomolecules the compounds studied would be able to participate by essentially their whole molecules, not just by some fragments of them.

### 2.4. Global Reactivity Parameters (GRP)

Analysis of the GRP values provided in Table 2 for both series of the compounds studied shows the following. (i) The compounds of both series have quite high IP values, 6.15–6.43 eV and 6.08–6.42 eV for the series SM-IMP and DA, respectively. The EA values for the series SM-IMP-01–SM-IMP-13 are not very high, being within 1.53–1.87 eV, whereas for the series DA-01–DA-05 they are noticeably higher, being within 2.07–3.18 eV. In the series SM-IMP-01–SM-IMP-13, the highest IP value, 6.43 eV, belongs to the compound SM-IMP-03, with two fluorine, and the lowest IP values, 6.15 eV, belong to the compounds SM-IMP-08 and SM-IMP-09, which bear just one Br substituent; the highest EA values in this series, 1.87 eV, belong to the compounds SM-IMP-01, without any substituents in the phenyl ligands, and SM-IMP-05, with F- and Cl substituents in the phenyl ligand, whereas the lowest EA value, 1.53 eV, is characteristic of the compound SM-IMP-13, with one Br substituent and alkyl and phenyl ligands attached to the amido link. In the series DA-01–DA-05 the highest IP and EA values, 6.42 and 3.18 eV, respectively, characterize the compound DA-05 with a nitro group, and the lowest IP and EA values, 6.08 and 2.07 eV, respectively, characterize the compound DA-04 with a methoxy group. Generally, all compounds considered should be relatively poor electron donors but quite good electron acceptors, especially the compounds of the DA series, more specifically, DA-05. (ii) The global electronegativity *X* along with global electrophilicity *ω* have noticeably high values, 3.875–4.145 eV and 3.202–3.861 eV, respectively, for the series SM-IMP-01–SM-IMP-13, and 4.075–4.8 eV and 4.141–7.111 eV, respectively, for series DA-01–DA-05. This supports the suggestion of these compounds being quite good electron acceptors in general. In the series SM-IMP-01–SM-IMP-13, the highest *X* value, 4.145 eV, belongs to the compounds SM-IMP-01, without any substituents in the phenyl ligands, and the lowest *X* value, 3.875 eV, characterizes the compound SM-IMP-13, with one Br substituent and alkyl and phenyl ligands attached to the amido link. Further, in this series the highest *ω* value, 3.861 eV, again characterizes the compound SM-IMP-01, and the lowest *ω* value, 3.202 eV, characterizes the compound SM-IMP-13. In the series DA-01–DA-05, the highest values of both parameters, 4.8 and 7.111 eV, are characteristic of the compound DA-05, which is in line with its highest EA value. The lowest values of these two parameters, 4.075 and 4.141 eV, respectively, again characterize the compound DA-04. Thus, again, generally, all compounds considered should be quite good electron acceptors, especially the compounds of the DA series, and more specifically, DA-05. (iii) The global chemical hardness values for the series SM-IMP-01–SM-IMP-13 are relatively close to each other, being within 2.215–2.345 eV, and the same can be said about the global softness values, which are noticeably lower than the global hardness values, varying within 0.213–0.226 eV. The highest value of the global hardness and the lowest value of the global softness, 2.345 and 0.213 eV, respectively, belong to the compound SM-IMP-13, thus characterizing it as the least reactive in the series. The lowest value of global hardness and the highest value of global softness, 2.215 and 0.226 eV, respectively, characterize the compound SM-IMP-02, thus, it is the most reactive compound in the series. In the series DA-01–DA-05, the global hardness values are in general lower than in the previous series, being within 1.62–2.085 eV, and the global softness values are higher, being within 0.240–0.309 eV. Therefore, these compounds are in general more reactive than the SM-IMP compounds. The highest global hardness value and the lowest global softness value, 1.62 and 0.309 eV, respectively, are the characteristics of the compound DA-05, thus making it the most reactive species in both series.

### 2.5. Molecular Electrostatic Potential Plots

**Figure 4:**
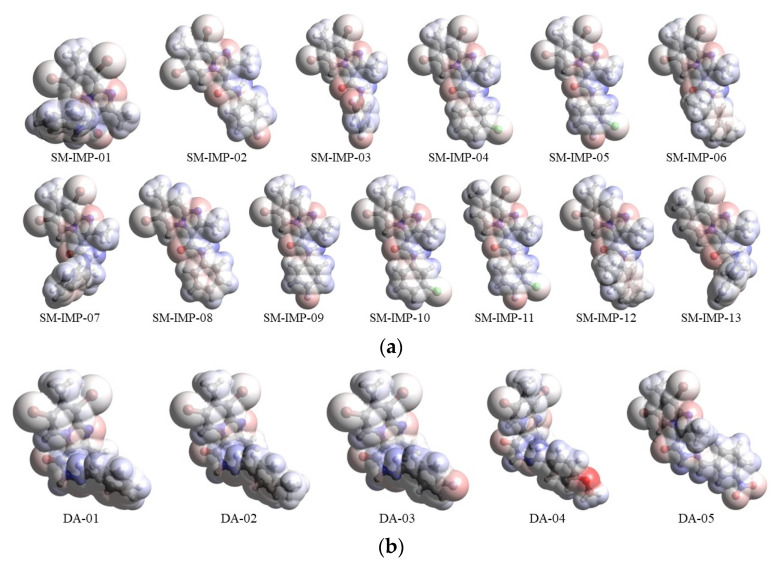
Molecular electrostatic potential (MEP) maps for the investigated compounds: series SM-IMP-01–SM-IMP-13 (**a**) and DA-01–DA-05 (**b**), B3LYP/6-311+G(d,p), implicit water.

Analysis of the MEP plots (Figure 4) for both series shows that in molecules of the compounds studied, there are areas of accumulation of both negative (as indicated by red color) and positive (as indicated by blue color) electrostatic potential. Negative potential accumulates on more electronegative atoms such as oxygen, nitrogen, and fluorine, to less extent on chlorine, and very little on bromines, if anything at all. In addition, some negative potential accumulation can be seen inside or on top of phenyl rings. Positive potential accumulation can be seen predominantly on hydrogens of alkyl, phenyl, and amino groups. These results imply that the compounds of both series can form various electrostatic and dispersion interactions and hydrogen bonds with polar solvent molecules and, much more important, with biomolecules, thus forming various complexes with proteins, etc. Additionally, the results imply that these compounds can participate in various chemical reactions both as electrophiles and nucleophiles.

### 2.6. Antimicrobial Activity of the Substituted Imidazopyridines (IMPs) (SM-IMP-01 to SM-IMP-13, and DA-01-05)

**Figure 5:**
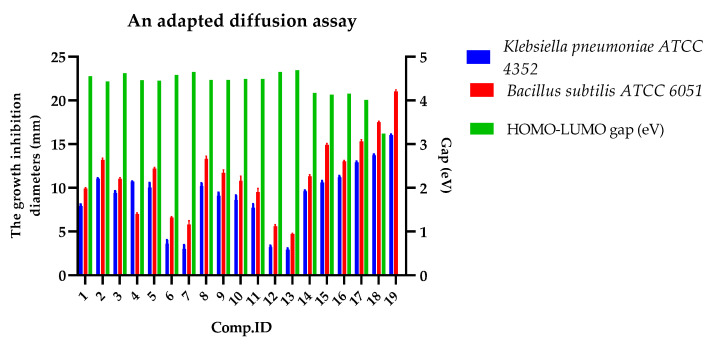
Antimicrobial assay results (qualitative) for tested compounds **1**–**18** as (SM-IMP-01) to SM-IMP-13), and (DA-01-05), respectively. Wherein, 19 was used as a standard drug, ciprofloxacin.

In order to see the antimicrobial actions of synthesized compounds, we subjected them to analysis with two different approaches: first with qualitative testing and second with quantitative determinations. The qualitative results obtained are displayed in Figure 5 (Table S1). The results from qualitative analysis suggested that there is an obvious influence of halogen (-Br) at 6 and 8 positions on bromo-Imidazo[1,2-a]Pyridine-3-Carboxamide series (Figure 5). Thus, we used to compare the effect of -Br substitutions on scaffold Imidazo[1,2-a]Pyridine-3-Carboxamide (IMPCs). In the first series, upto SM-IMP-01 to SM-IMP-07, we can see that antimicrobial activity (zone of inhibitions (ZOI) for both of bacterial strains) was influenced by the higher electronegative group, -F (fluorine) (with 2 -Br groups). Thus, compound SM-IMP-02 showed the highest ZOI at 11 mm at 100 µg/mL against *Klebsiella pneumoniae* ATCC 4352. Wherein, compounds with electron realizing groups such as -CH_3_ and -C_2_H_5_ groups depicted lesser ZOI, indicating lesser potencies against selected strains of bacteria, e.g., compound SM-IMP-06 and SM-IMP-07 were demonstrated ZOIs as 3.6 and 3 mm diameters, respectively, against *Klebsiella pneumoniae* ATCC 4352 (Figure 5). Coming to another series with 6-bromo-Imidazo[1,2-a]Pyridine-3-Carboxamides (only one -Br on Imidazopyridine scaffold, only at six positions), we noticed that for the same substituents of aldehydes, there was significant role missing of -Br group at eighth position, as seen by lesser ZOI values. It also means that among the two aforementioned series, compounds with the addition -Br group at the eighth position would act as better antibacterial agents. Furthermore, additions of halogens on the terminal aryl (-Ar) group attached to (IMP-C=O-NH---) functionality increases the bioactivity of compounds. This trend can easily be observed from concerned HOMO-LUMO energy gaps. In the last series, wherein, we had five hydrazides of IMPCs, they tend to have lesser HOMO-LUMO energy gaps, and also showed higher antimicrobial activities when tested with adapted agar diffusion assays. The lesser energy gaps for hydrazides (DA-01-DA-05) explain the fact that electronic behaviors of compounds would likely have associations with enhancements or decrements in bioactivities. From our qualitative antimicrobial assays, among all 18 compounds, DA-05 (which also had a lesser energy gap: 3.24 eV) was found to have a better zone of inhibition values as 13.7 mm and 17.5 mm against *Klebsiella pneumoniae ATCC 4352* and *Bacillus subtilis ATCC 6051*, respectively (Figure 5). 

**Figure 6:**
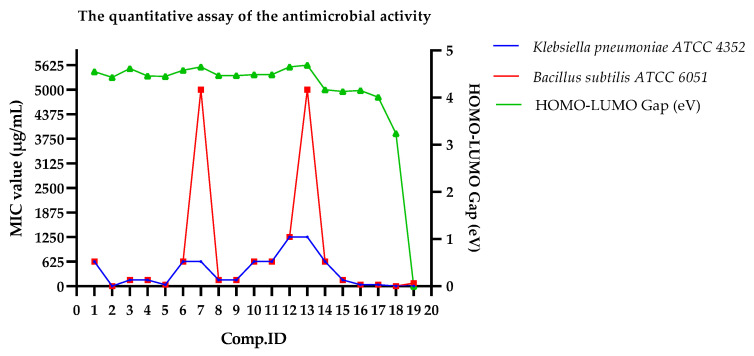
The results of the quantitative assay of the antimicrobial activity of tested compounds **1**–**18** as (SM-IMP-01) to SM-IMP-13), and (DA-01-05), respectively. Wherein, 19 was used as a standard drug, ciprofloxacin, and the corresponding MIC value (μg/mL).

In order to have a better understanding of antimicrobial activities exhibited by compounds, we tested all compounds with the quantitative assay and reported their corresponding MIC values (μg/mL) (Figure 6) (Table S2). From analysis, we found that among two series, wherein there is a role of -Br group on scaffold Imidazo[1,2-a]Pyridine-3-Carboxamide (SM-IMP-01 to SM-IMP-13), compounds with -Br at the eighth position demonstrated lesser MIC values than compounds with -H at the eighth position on imidazopyridine ring (Figure 6). Additionally, compounds with added halogens and having -Br at the eighth position on the IMP ring were found to be better antibacterial agents with minimum inhibitory concentrations required. This can be seen by comparing any two compounds, for example, compound SM-IMP-02 and SM-IMP-09 (MICs of 4.8 µg/mL and 156 µg/mL, respectively, tested against *Klebsiella pneumoniae* ATCC 4352) (Figure 6). Among hydrazide series (DA-01-DA-05), compounds with -NO_2_ substituent, i.e., DA-05 showed a lesser MIC value of 4.8 µg/mL against each tested bacterial strain, *Klebsiella pneumoniae* ATCC 4352 and *Bacillus subtilis* ATCC 6051. Among all 18 compounds, DA-05 had the lowest MIC value recorded as 4.8 µg/mL, and also had the lowest lesser energy gap: 3.24 eV. We also noticed one fact that compound SM-IMP-13, which had the highest energy gap of 4.69 eV showed poor antibacterial potencies in both qualitative and quantitative assays (Figure 6). This compound also has electron-releasing groups at terminal aryl moiety and thus, it is definite that the electronic behavior of the compound would likely be responsible for tested bioactivities. For both qualitative and quantitative assays, we used ciprofloxacin as a positive control. 

#### Relationship of Biological Assay with DFT Properties

If we compare chemical hardness (*η*) values of the most active (MIC: 4.8 µg/mL, against *K. pneumoniae ATCC 4352*) and less active (MIC: 1250 µg/mL, against *K. pneumoniae ATCC 4352*) (in terms of antimicrobial activities, except hydrazide series molecules) molecules SM-IMP-02 and SM-IMP-13, respectively, we can see that former compound has lesser hardness (2.215 eV) than the later one (2.345 eV). Further, when we talk about their softness values (S), we observed that the most active molecule (in terms of antibacterial assays), had higher softness (S: 2.26 eV) than the less bioactive molecules SM-IMP-13 (S: 0.213 eV). In terms of the hydrazide series, molecule DA-05 had a higher softness and fewer hardness values among all series, which also explains the highest bioactivities among all 18 compounds. Further, based on the bioactivity and relatively high HOMO/LUMO gap of SM-IMP-02, and SM-IMP-13, the consideration of the HOMO/LUMO gap is important when we wish to see a possible relationship amongst DFT properties and bioactivity for most active and poor active molecules (among Imidazopyridine carboxamide series, except hydrazides). We were selecting these molecules as a representative to establish the relations among calculated DFT properties and bioactivities. However, one can take other molecules to explain this trend also. The importance of chemical hardness and softness values with respect to increments or decrements in biological activities (specifically, antimicrobial actions) are well explained and referred to from the previous literature reports [4,40,41].

### 2.7. Spectral Data of Synthesized Compounds

The recording probe temperature and the number of scans for NMR analysis were as follows:

(SM-IMP-01) to (SM-IMP-13) except (SM-IMP-08): probe temperature: 300.0 K and numbers of scans: 16 for ^1^H-NMR; (SM-IMP-08): probe temperature: 300.0 K and numbers of scans: 512; for ^13^C-NMR analysis, a minimum of 1000 scans were maintained and Temp_Get was at ≈20 °C as recorded with Jeol, USA, NMR machine (for further details on NMR parameters, please refer supporting information). NMR data for (SM-IMP-01) to (SM-IMP-13) are coherent with earlier reported data and are available in supporting information. However, NMR, FTIR, and mass data for new molecules are as below:

(E)-N′-benzylidene-6,8-dibromo-2,7-dimethylimidazo [1,2-a]pyridine-3-carbohydrazide (DA-01): ^1^H-NMR (400 MHz, DMSO-d_6_) δ 9.50 (s, 1H), 7.75 (s, 1H), 7.54–7.41 (m, 2H), 7.36 (s, 1H), 7.32–7.26 (m, 2H), 2.70–2.66 (m, 3H), 2.40–2.36 (m, 3H); elemental analysis cald. For C_17_H_14_Br_2_N_4_O: C, 45.36; H, 3.14; N, 12.45; O, 3.55, found: C, 45.32; H, 3.11; N, 12.55; O, 3.90; ESI-MS *m*/*z*: 450.9 [M]^+^.

(E)-6,8-dibromo-2,7-dimethyl-N′-(4-methylbenzylidene)imidazo [1,2-a]pyridine-3-carbohydrazide (DA-02): elemental analysis cald. For C_18_H_16_Br_2_N_4_O: C, 46.58; H, 3.47; N, 12.07; O, 3.45, found: C, 46.41; H, 3.36; N, 12.00; O, 3.76; ESI-MS *m*/*z*: 465.0 [M + 1]^+^.

(E)-6,8-dibromo-N′-(4-fluorobenzylidene)-2,7-dimethylimidazo [1,2-a]pyridine-3-carbohydrazide (DA-03): elemental analysis cald. For C_17_H_13_Br_2_FN_4_O: C, 43.62; H, 2.80; N, 11.97; O, 3.42, found: C, 43.61; H, 2.78; N, 11.92; O, 3.41; ESI-MS *m*/*z*: 468.9 [M]^+^.

(E)-6,8-dibromo-N′-(4-methoxybenzylidene)-2,7-dimethylimidazo [1,2-a]pyridine-3-carbohydrazide (DA-04): elemental analysis cald. For C_18_H_16_Br_2_N_4_O_2_: C, 45.03; H, 3.36; N, 11.67; O, 6.66, found: C, 45.01; H, 3.27; N, 11.53; O, 6.74; ESI-MS *m*/*z*: 481.0 [M + 1]^+^.

(E)-6,8-dibromo-2,7-dimethyl-N′-(3-nitrobenzylidene)imidazo [1,2-a]pyridine-3-carbohydrazide (DA-05): elemental analysis cald. For C_17_H_13_Br_2_N_5_O_3_: C, 41.24; H, 2.65; N, 14.14; O, 9.69, found: C, 41.21; H, 2.61; N, 14.16; O, 9.77; ESI-MS *m*/*z*: 496.0 [M + 1]^+^.

## 3. Materials and Methods

### 3.1. General Information

For current work, all chemicals and solvents have been purchased from Sigma-Aldrich and Lab India, Pvt., Ltd., Mumbai. Reactants, (SM-1a-SM-1b) (dibromo and mono-bromo substituted 2-amino-γ-picoline) were procured from Lab India, Pvt., Ltd., Mumbai. The progress of reactions was monitored using the TLC (thin layer chromatography) plates procured from Merck and of silica-F254 coated aluminum plates category. Melting points reported herein were measured using “OptiMelt” melting point apparatus and were uncorrected. For proton (^1^H) and ^13^carbon (^13^C) nuclear magnetic resonance (NMR), we used Bruker (400 MHz) or JEOL, Japan; JNM ECZ400S/LI (400 MHz) instruments with tetramethylsilane (TMS) as internal standard and expressed for chemical shifts (the delta scale as parts per million (ppm)). For infrared spectral data (the range 4000–400 cm^−1^), we used “a Perkin Elmer, Waltham, MA, USA; Froner” Instrument, wherein for measuring elemental analyses of all compounds, we used “Elemental Analyzer, Hesse, Germany; Vario EL III (C, H, N, S, O) analyzer”. For mass analyses, we used “ThermoScienfic, Waltham, MA, USA; Ultimate 3000” instrument.

### 3.2. Chemistry

**Scheme 1:**
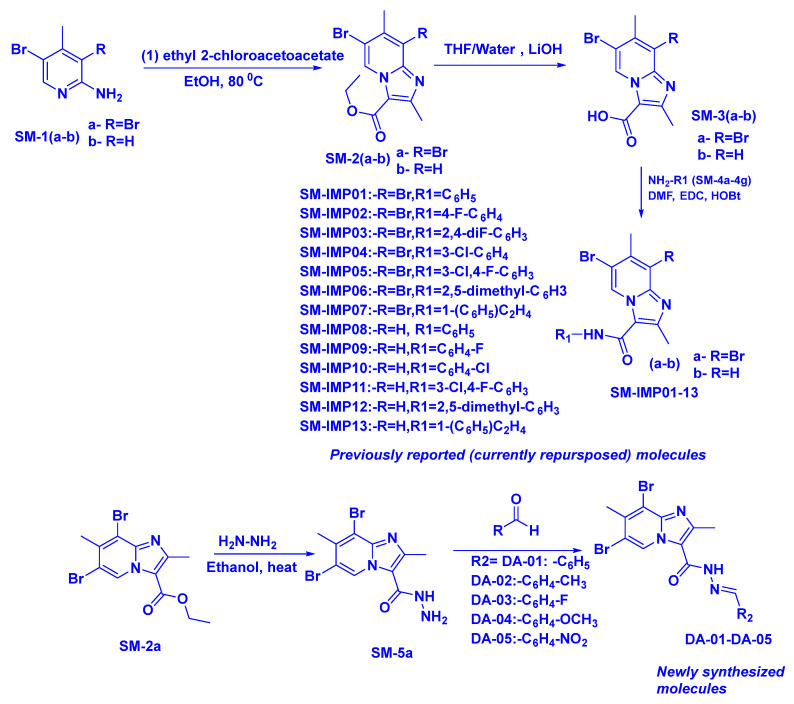
Reaction scheme for preparation of compounds (SM-IMP-01) to (SM-IMP-13) and (DA-01-DA-05).

Scheme 1 was adopted from the synthetic route reported in the earlier literature and elaborates on the synthesis protocol for molecules (SM-IMP-01) to (SM-IMP-13) [26]. Scheme 1 also details hydrazides (DA-01-DA-05). Reactants, (SM-1a-SM-1b) (dibromo and mono-bromo substituted 2-amino-γ-picoline) were reacted with ethyl 2-chloroacetoacetate (1) in the presence of base to yield desired ethyl imidazo [1,2-a]pyridine-3-carboxylates (SM-2a-SM-2b). Further, imidazo [1,2-a]pyridine-3-carboxylic acids (SM-3a-SM-3b) were achieved via hydrolysis of esters (SM-2a-SM-2b). These acid derivatives were then treated with substituted anilines (SM-4a-f) or ɑ-methylbenzylamine (SM-4g) using hydroxybenzotriazole (HOBt) as coupling agent to afford substituted 2,7-dimethylimidazo [1,2-a]pyridine-3-carboxamides (SM-IMP-01 to SM-IMP-13). Further, we aimed for the synthesis of hydrazides (DA-01 to DA-05). For this, desired ethyl imidazo [1,2-a]pyridine-3-carboxylate (SM-2a) was treated with hydrazine hydrate in presence of ethanol as a solvent and heated this mixture to 80 °C in a round bottom flask (RBF), which then yielded corresponding hydrazine (SM-5a). The hydrazine, (SM-5a) was then coupled with varieties of aromatic aldehydes resulting final carbohydrazide (DA-01 to DA-05). For DA-02, DA-03, and DA-04, we used *p*-substituted aromatic aldehyde; wherein for DA-05, it was *m*-substituted aromatic aldehyde (please refer to Figures S1–S60 for spectral data).

### 3.3. Theoretical Analyses

Density functional theory (DFT) studies were performed using the Gaussian 16 software [27]. We optimized the structures of all compounds studied without any symmetry constraints and then performed frequency calculations to verify that the optimized structures are true energy minima. All calculations were performed with the hybrid density functional B3LYP [28] and the triple-zeta split-valence polarized basis set 6-311+G* [29,30] (with one set of polarization and one set of diffuse functions on heavier atoms), the approach further referred to as B3LYP/6-311+G*. The studies were completed with the B3LYP/6-311+G* approach with the implicit effects from water (dielectric constant ε = 78.3553) taken into account, employing the self-reliable IEF-PCM approach [31] with the UFF default model as implemented in the Gaussian 16 software, with the electrostatic scaling factor α = 1.0.

Below, we consider the structures and energetics, natural population analysis (NPA) charges [32], frontier molecular orbitals (FMOs), and molecular electrostatic potential (MEP) maps for all compounds obtained with the implicit effects from water. Additionally, we used the values of the energies of highest occupied molecular orbital (HOMO)–lowest unoccupied molecular orbital (LUMO) to compute the global reactivity parameters (GRPs) [33,34,35,36,37,38,39,40,41] (Equations (1)–(6)). 

The chemical reactivity indices such as chemical hardness (*η*), electronegativity (*χ*), electronic chemical potential (*μ*), and electrophilicity Index (*ω*), were also calculated for all the synthesized molecules. The stability and reactivity of a chemical system are represented by chemical hardness (*η*). This descriptor is used as a measure of resistance to change in the electron distribution or charge in a molecule [4,39,40,41]. Electronegativity is given by expression *χ*, which is defined as the power of an atom in a molecule to attract electrons toward it [4,41]. The negative term of electronegativity of a molecule is defined as chemical potential [4,41]. Electrophilicity index (*ω*), is a measure of the propensity or capacity of a species to accept electrons introduced by Parr and is calculated using the electronic chemical potential and chemical hardness [40,41]. 

Equations (1) and (2) were used to calculate the values of the ionization potential (*IP*) and electron affinity (*EA*):

*IP* = −*E*_HOMO_ (1)

*EA* = −*E*_LUMO_ (2)

For global hardness *η* and electronegativity *χ* values we used Equations (3) and (4):

(3) $$\eta =\frac{\left[IP-EA\right]}{2}=\frac{\left[E_{LUMO}-E_{HOMO}\right]}{2}$$

(4) $$\chi =\frac{\left[IP+EA\right]}{2}=-\frac{\left[E_{LUMO}+E_{HOMO}\right]}{2}$$

Additionally, global electrophilicity *ω* value was calculated by Equation (5):

(5) $$\omega =\mu^{2}/2\eta$$

where $\mu =\frac{\left[E_{HOMO}+E_{LUMO}\right]}{2}$ is the chemical potential of the system.

Finally, the global softness *σ* value was computed with Equation (6):

(6) σ=1/2η

Open GL version of Molden 5.8.2 visualization program was used for the visualization of the structures and FMOs of the studied compounds [36], and Avogadro, version 1.1.1, was used to visualize the molecular electrostatic potential (MEP) maps [37,38].

### 3.4. Bioactivity Analyses

The in vitro antibacterial evaluation against *Klebsiella pneumoniae ATCC 4352* (Gram Negative Bacteria) and *Bacillus subtilis ATCC 6051* was carried out using qualitative and quantitative assays were carried out as per previously published protocol [39] (Please refer Supplementary Materials).

## 4. Conclusions

To summarize, we have synthesized 18 imidazopyridine-based compounds. Out of 18 compounds, we tried repurposing the 13 previously published compounds by testing them against *Klebsiella pneumoniae ATCC 4352* and *Bacillus subtilis ATCC 6051*. We further elaborated full accounts on various DFT parameters in order to assess chemical reactivity and stabilities. Such electronic behaviors of compounds can have a good relationship with biological activity and the trend can be seen from results obtained from qualitative and quantitative antimicrobial assays. Among all 18 synthesized compounds, compounds with a hydrazide core demonstrated narrower MIC values and also had lesser energy gaps, thus would better act as antibacterial agents than compounds with no hydrazide moiety in them. Such importance of the hydrazide core is also known from the prior literature reports available for hydrazide compounds. Thus, considering the full potential of IMPs, one can test out these moieties for further higher in vitro or in vivo models in order to develop safer and more potent antibacterial agents.

## Figures and Tables

**Table 1:** Electronic energies without (E_0_) and with zero-point (E_0_ + ZPE) corrections, HOMO and LUMO energies (E_(HOMO/LUMO)_), HOMO/LUMO gaps (DE_(HOMO/LUMO)_, and time-dependent DFT calculated (TDDFT) gaps, B3LYP/6-311+G(d,p), with implicit effects from water.

Compound	E_0_, A.U.	E_0_ + ZPE, A.U.	E_(HOMO/_ _LUMO)_, A.U.	DE_(HOMO/_ _LUMO)_, eV	TDDFT Gap, eV
SM-IMP-01	−6005.551842	−6005.293120	−0.23600/−0.06880	4.55	3.95
SM-IMP-02	−6104.828771	−6104.577934	−0.22966/−0.06689	4.43	3.92
SM-IMP-03	−6204.092300	−6203.850115	−0.23619/−0.06648	4.62	4.09
SM-IMP-04	−6465.183041	−6464.933668	−0.23269/−0.06869	4.46	3.92
SM-IMP-05	−6564.446170	−6564.205187	−0.23207/−0.06865	4.45	3.91
SM-IMP-06	−6084.213075	−6083.900065	−0.23289/−0.06456	4.58	4.07
SM-IMP-07	−6084.204338	−6083.889031	−0.23353/−0.06250	4.65	4.10, 4.22
SM-IMP-08	−3432.025895	−3431.756301	−0.22610/−0.06173	4.47	3.94
SM-IMP-09	−3531.294066	−3531.032988	−0.22603/−0.06189	4.47	3.94
SM-IMP-10	−3891.648466	−3891.388547	−0.22936/−0.06435	4.49	3.93
SM-IMP-11	−3990.912017	−3990.660204	−0.22852/−0.06370	4.49	3.94
SM-IMP-12	−3510.678376	−3510.354618	−0.22928/−0.05854	4.65	4.11
SM-IMP-13	−3510.670015	−3510.344084	−0.22852/−0.05634	4.69	4.15
DA-01	−6098.995509	−6098.715663	−0.23274/−0.07953	4.17	3.49
DA-02	−6138.324011	−6138.016908	−0.23020/−0.07854	4.13	3.46
DA-03	−6198.264953	−6197.993317	−0.23282/−0.08043	4.15	3.46
DA-04	−6213.555413	−6213.243263	−0.22354/−0.07633	4.01	3.41
DA-05	−6303.573759	−6303.291645	−0.23588/−0.11688	3.24	3.16, 3.65

**Table 2:** GRPs for all the compound studies, eV, B3LYP/6-311+G(d,p), implicit water.

Compound	IP	EA	Gap	*X*	*η*	*μ*	S	*ω*
SM-IMP-01	6.42	1.87	4.55	4.145	2.275	−4.145	0.220	3.861
SM-IMP-02	6.25	1.82	4.43	4.035	2.215	−4.035	0.226	3.675
SM-IMP-03	6.43	1.81	4.62	4.12	2.310	−4.12	0.216	3.674
SM-IMP-04	6.33	1.87	4.46	4.10	2.230	−4.10	0.224	3.769
SM-IMP-05	6.31	1.86	4.45	4.085	2.225	−4.085	0.225	3.750
SM-IMP-06	6.34	1.76	4.58	4.05	2.29	−4.05	0.218	3.581
SM-IMP-07	6.35	1.70	4.65	4.025	2.325	−4.025	0.215	3.484
SM-IMP-08	6.15	1.68	4.47	3.915	2.235	−3.915	0.224	3.429
SM-IMP-09	6.15	1.68	4.47	3.915	2.235	−3.915	0.224	3.429
SM-IMP-10	6.24	1.75	4.49	3.995	2.245	−3.995	0.223	3.555
SM-IMP-11	6.22	1.73	4.49	3.975	2.245	−3.975	0.223	3.519
SM-IMP-12	6.24	1.59	4.65	3.915	2.325	−3.915	0.215	3.296
SM-IMP-13	6.22	1.53	4.69	3.875	2.345	−3.875	0.213	3.202
DA-01	6.33	2.16	4.17	4.245	2.085	−4.245	0.240	4.321
DA-02	6.26	2.13	4.13	4.195	2.065	−4.195	0.242	4.261
DA-03	6.34	2.19	4.15	4.265	2.075	−4.265	0.241	4.383
DA-04	6.08	2.07	4.01	4.075	2.005	−4.075	0.249	4.141
DA-05	6.42	3.18	3.24	4.8	1.62	−4.8	0.309	7.111

## Data Availability

Data used herein in this research is a part of ongoing PhD. work and will be made available as appropriate.

## Supplementary Materials

The following supporting information can be downloaded at: https://www.mdpi.com/article/10.3390/molecules28062801/s1. Figures S1–S60: H-NMR, 13C-NMR, FTIR, Mass spectral data for compounds SM-IMP-01 to DA-05. Figure S61: The results of the quantitative assay of the antimicrobial activity of tested compounds 1–18 as (SM-IMP-01) to SM-IMP-13), and (DA-01-05), respectively. Wherein, 19 was used as a standard drug, ciprofloxacin, and the corresponding MIC value (μg/mL). Table S1: The results of the qualitative assay of the antimicrobial activity of the tested compounds by using an adapted diffusion assay (the growth inhibition diameters were measured and expressed in mm). Table S2: The results of the quantitative assay of the antimicrobial activity of tested compounds 1-18 as (SM-IMP-01) to SM-IMP-13), and (DA-01-05), respectively. Wherein, 19 was used as a standard drug, ciprofloxacin, and the corresponding MIC value (μg/mL).
