# Supplementary material for: Theoretical and Anti-Klebsiella pneumoniae Evaluations of Substituted 2,7-dimethylimidazo[1,2-a]pyridine-3-carboxamide and Imidazopyridine Hydrazide Derivatives
Source: Molecules. 2023 Mar 20;28(6):2801. doi: 10.3390/molecules28062801 (PMC10051578; doi:10.3390/molecules28062801)
Supplement: Supplementary file 1 [file molecules-28-02801-s001.zip › molecules-2271346-supplementary.pdf]

Supplementary Material

Theoretical and Anti-*Klebsiella pneumoniae* Evaluations of Substituted 2,7-dimethylimidazo[1,2-a]pyridine-3-carboxamide and Imidazopyridine Hydrazide Derivatives

Suraj N. Mali [1,*,†], Amit Anand [1,†], Magdi E. A. Zaki [2], Sami A. Al-Hussain [2,*], Rahul D. Jawarkar [3], Anima Pandey [1] and Aleksey Kuznetsov [4]

[1] Department of Pharmaceutical Sciences and Technology, Birla Institute of Technology, Ranchi 835215, India; anandamit1005@gmail.com (A.A.); apandey@bitmesra.ac.in (A.P.)
[2] Department of Chemistry, Faculty of Science, Imam Mohammad Ibn Saud Islamic University (IMSIU), Riyadh 11623, Saudi Arabia; mezaki@imamu.edu.sa
[3] Department of Medicinal Chemistry and Drug Discovery, Dr Rajendra Gode Institute of Pharmacy, University Mardi Road, Amravati 444603, India; rahuljawarkar@gmail.com
[4] Department of Chemistry, Universidad Técnica Federico Santa Maria, Santiago 7660251, Chile
* Correspondence: mali.suraj1695@gmail.com (S.N.M.); sahussain@imamu.edu.sa (S.A.A.-H.)
† These authors contributed equally to this work.

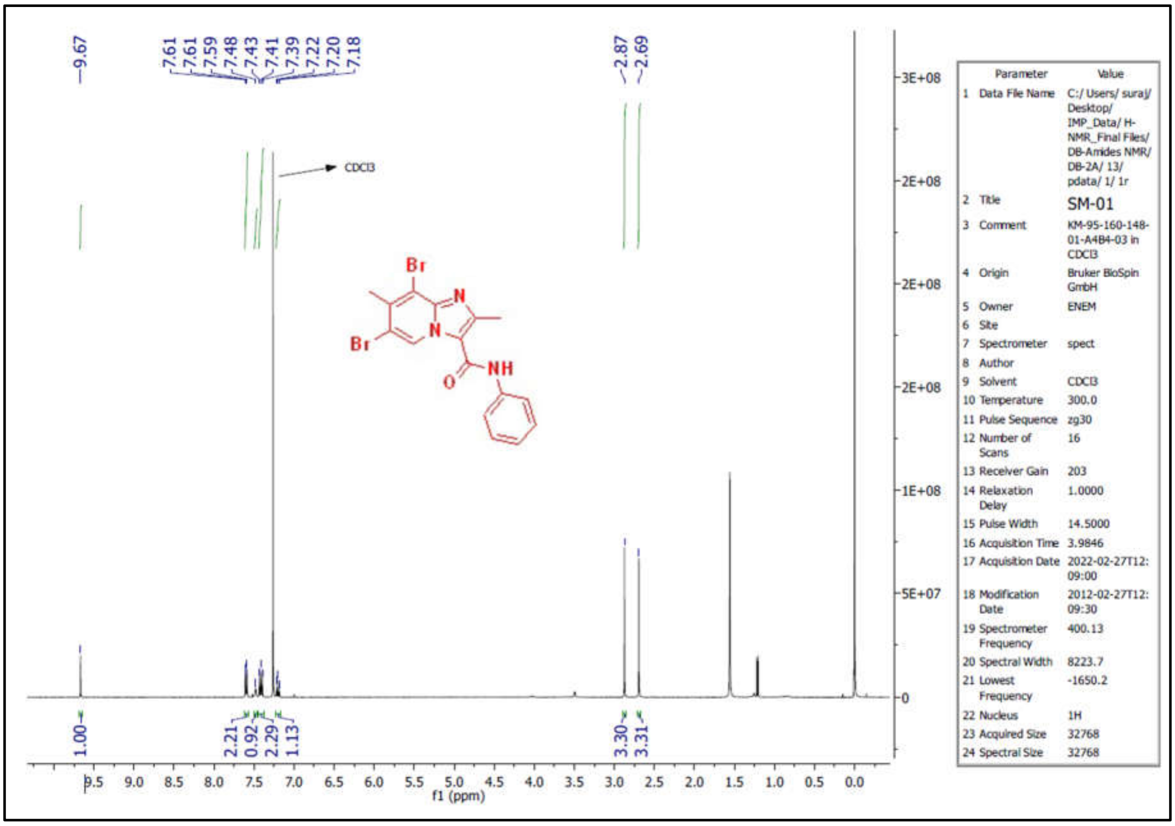

Figure S1. ^{1}H-NMR spectra of 6,8-dibromo-2,7-dimethyl-N-phenylimidazo[1,2-a]pyridine-3-carboxamide (**SM-IMP-01**).

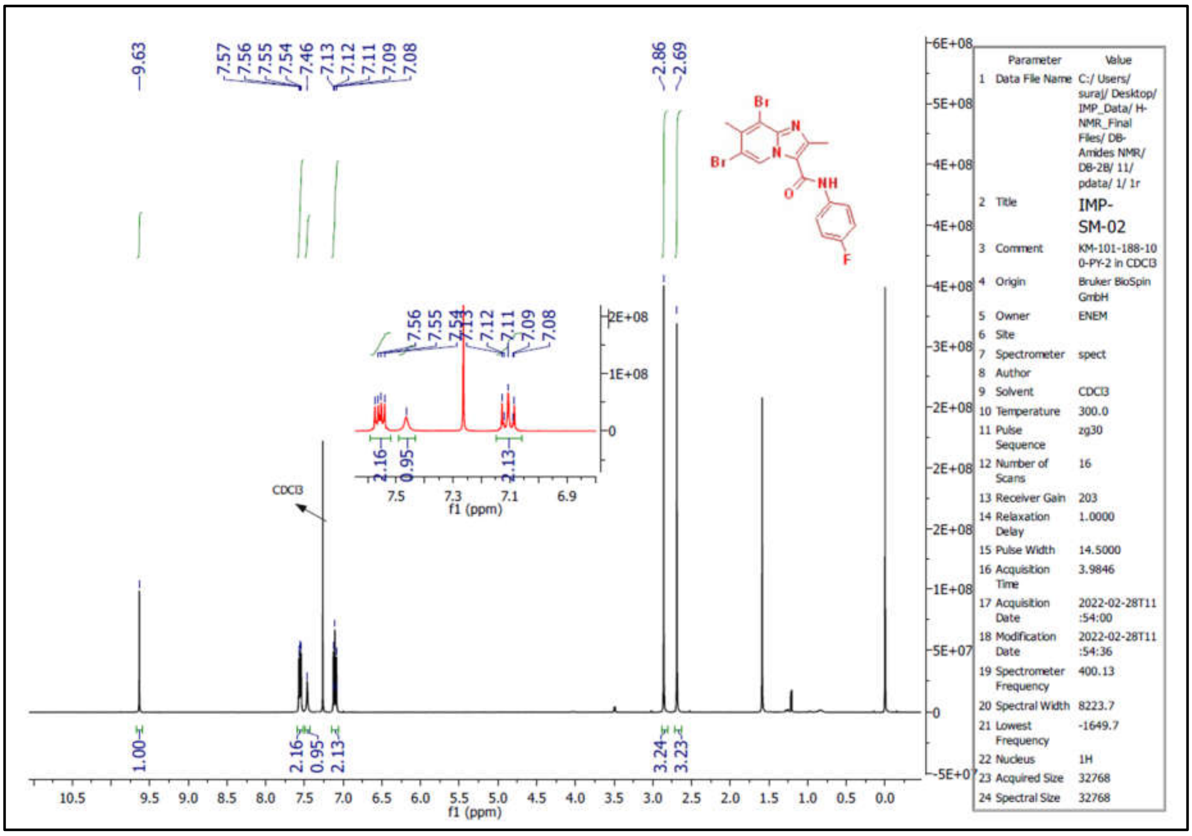

Figure S2. ^{1}H-NMR spectra of 6,8-Dibromo-2,7-Dimethyl-N-(4-Fluorophenyl)Imidazo[1,2-a]Pyridine-3-Carboxamide (**SM-IMP-02**).

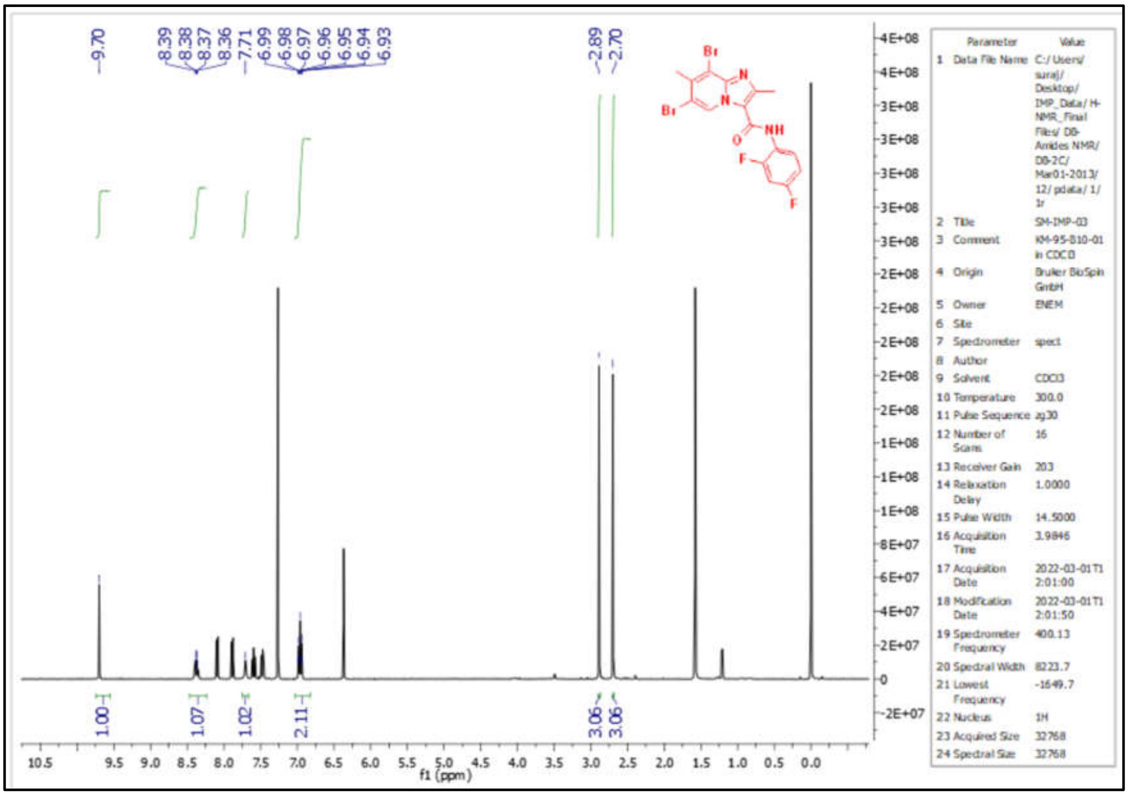

Figure S3. ^{1}H-NMR spectra of 6,8-Dibromo-N-(2,4-Difluorophenyl)-2,7-Dimethylimidazo[1,2-a]Pyridine-3-Carboxamide (**SM-IMP-03**).

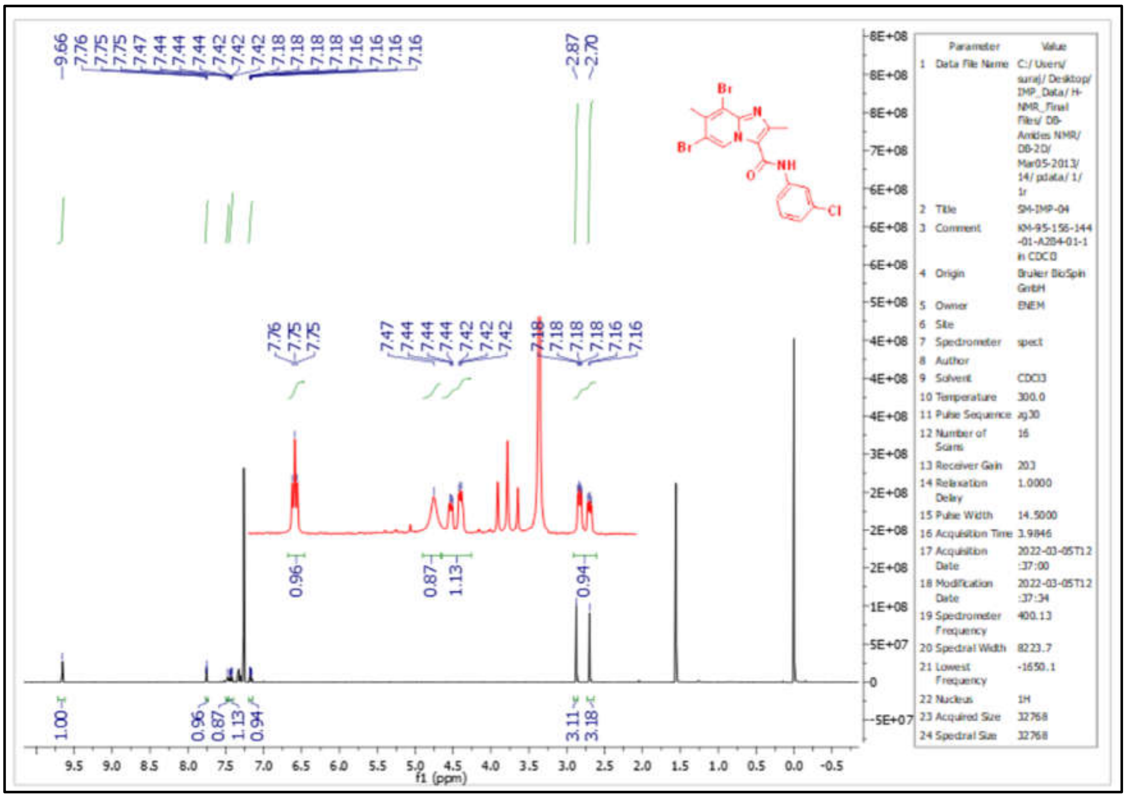

Figure S4. ^{1}H-NMR spectra of 6,8-Dibromo-N-(3-Chlorophenyl)-2,7-Dimethylimidazo[1,2-a]Pyridine-3-Carboxamide (**SM-IMP-04**).

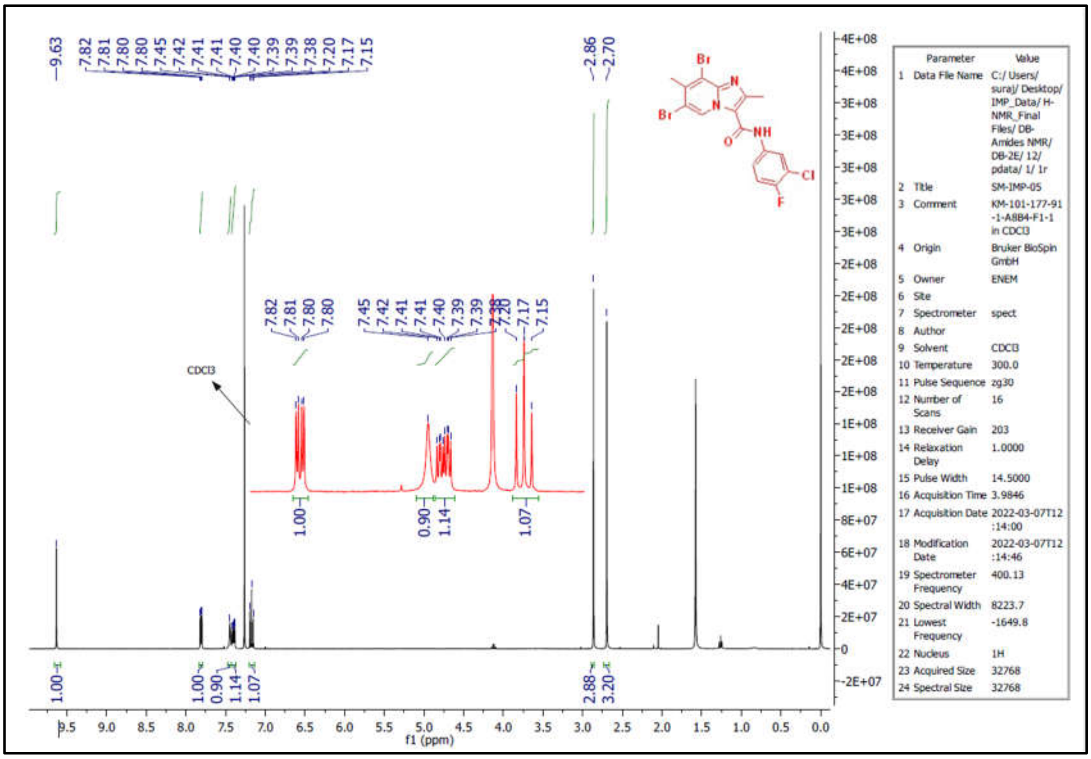

Figure S5. ^{1}H-NMR spectra of 6,8-Dibromo-N-(3-Chloro-4-Fluorophenyl)-2,7-Dimethylimidazo[1,2-a]Pyridine-3-Carboxamide (**SM-IMP-05**).

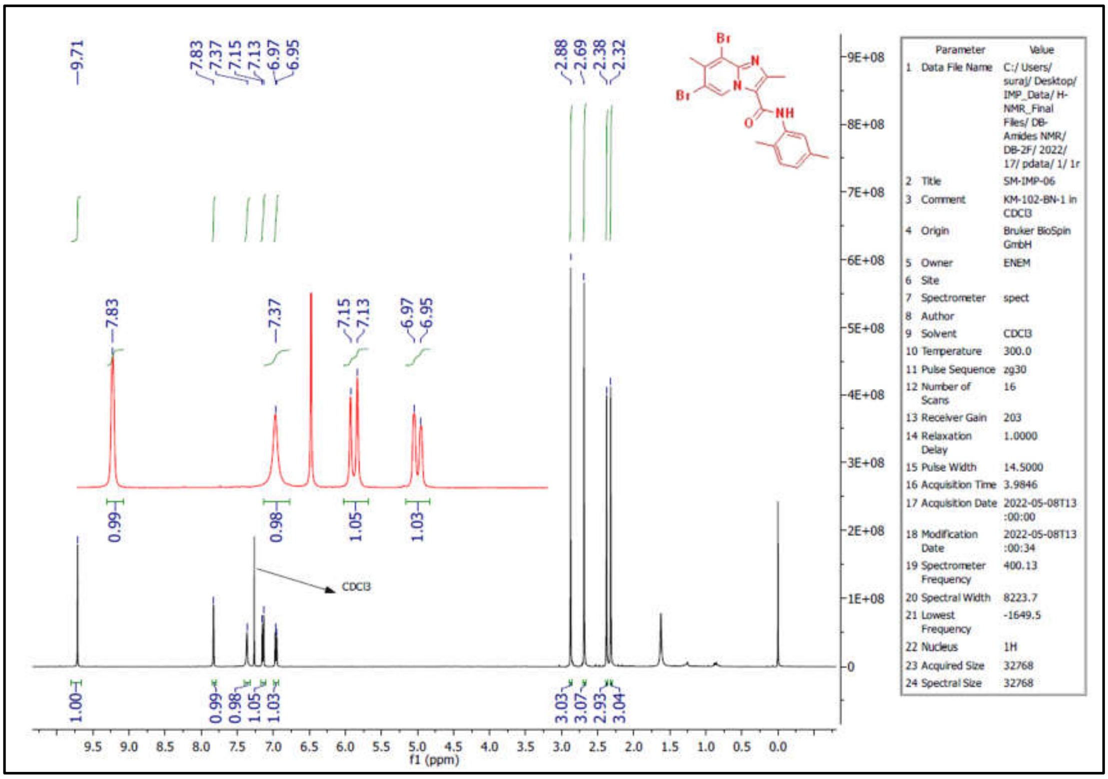

Figure S6. ^{1}H-NMR spectra of 6,8-Dibromo-N-(2,5-Dimethylphenyl)-2,7-Dimethylimidazo[1,2-a]Pyridine-3-Carboxamide (**SM-IMP-06**).

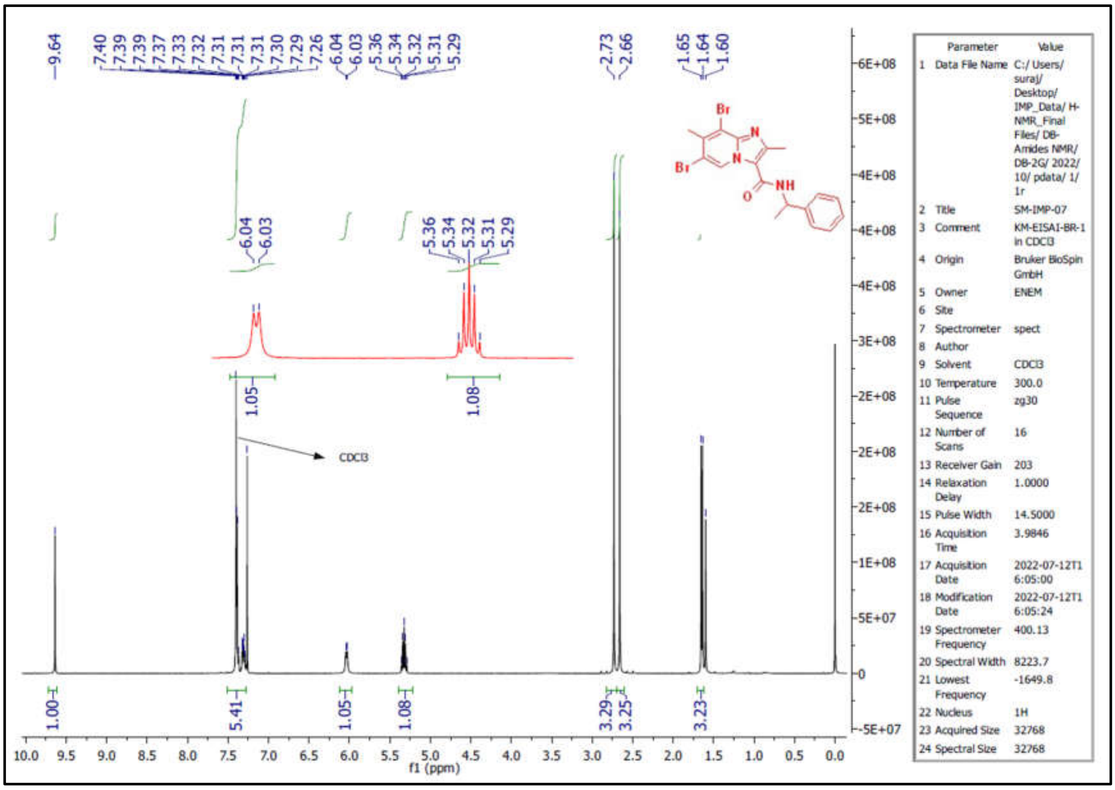

Figure S7. ^{1}H-NMR spectra of 6,8-Dibromo-2,7-Dimethyl-N-(1-Phenylethyl)Imidazo[1,2-a]Pyridine-3-Carboxamide (**SM-IMP-07**).

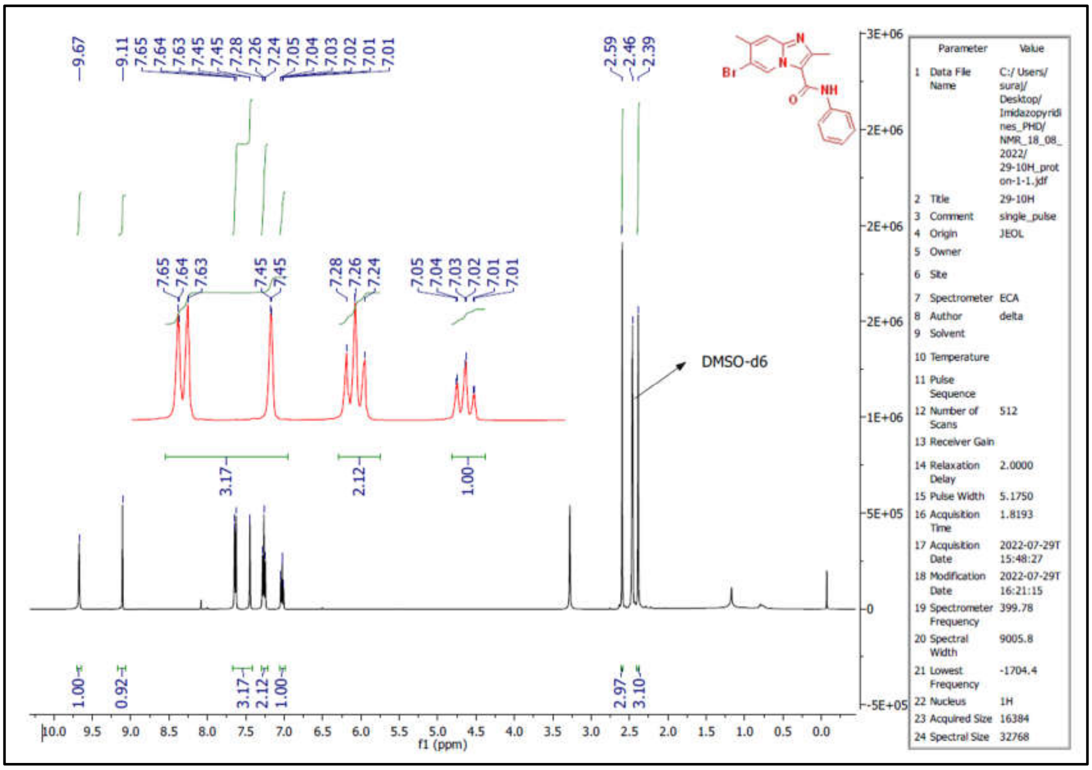

Figure S8. ^{1}H-NMR spectra of 6-Bromo-2,7-Dimethyl-N-Phenylimidazo[1,2-a]Pyridine-3-Carboxamide (**SM-IMP-08**).

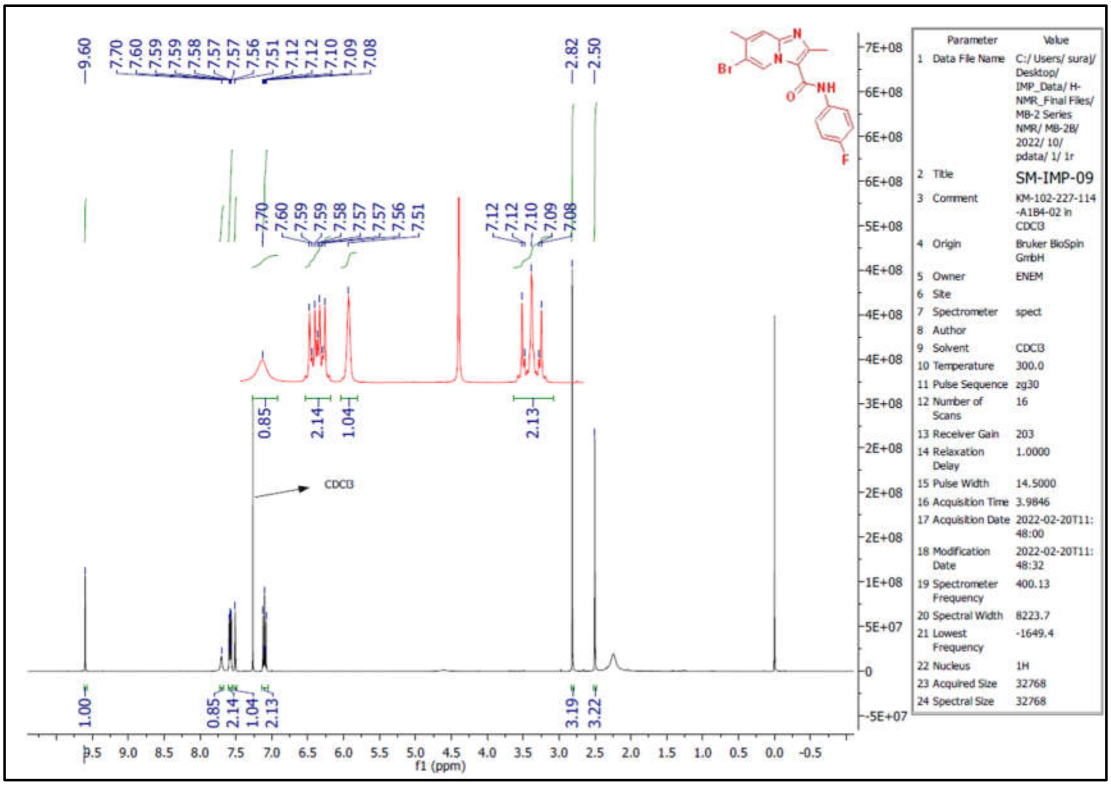

Figure S9. ^{1}H-NMR spectra of 6-Bromo-N-(4-Fluorophenyl)-2,7-Dimethyl Imidazo[1,2-a]Pyridine-3-Carboxamide (**SM-IMP-09**).

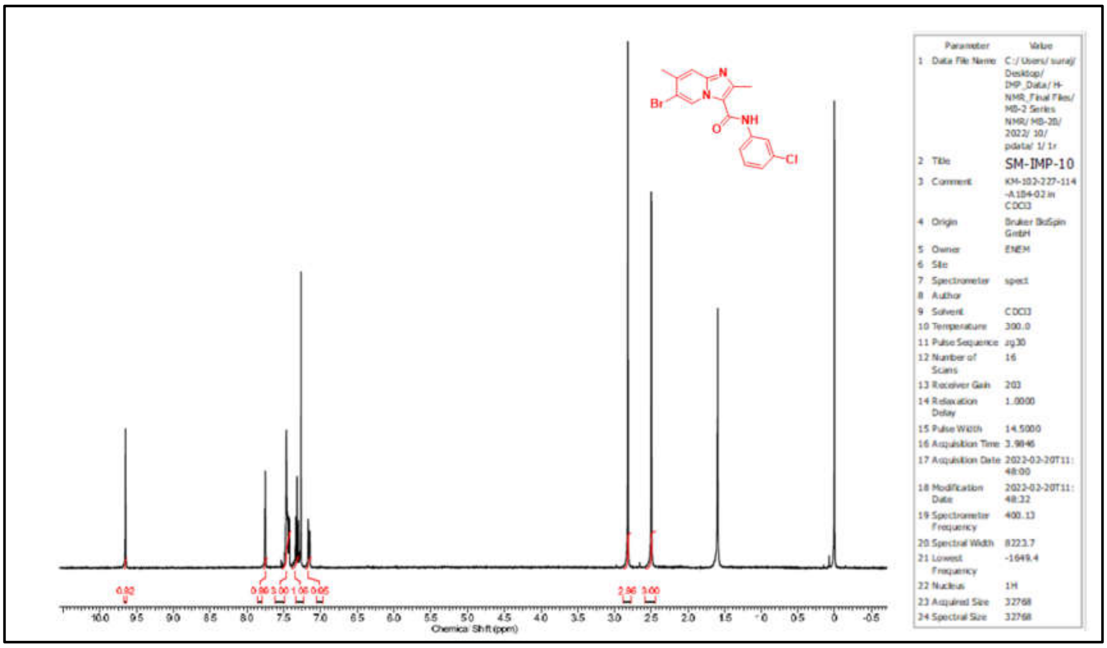

Figure S10. ^{1}H-NMR spectra of 6-Bromo-N-(3-Chlorophenyl)-2,7-Dimethyl Imidazo[1,2-a]Pyridine-3-Carboxamide (**SM-IMP-10**).

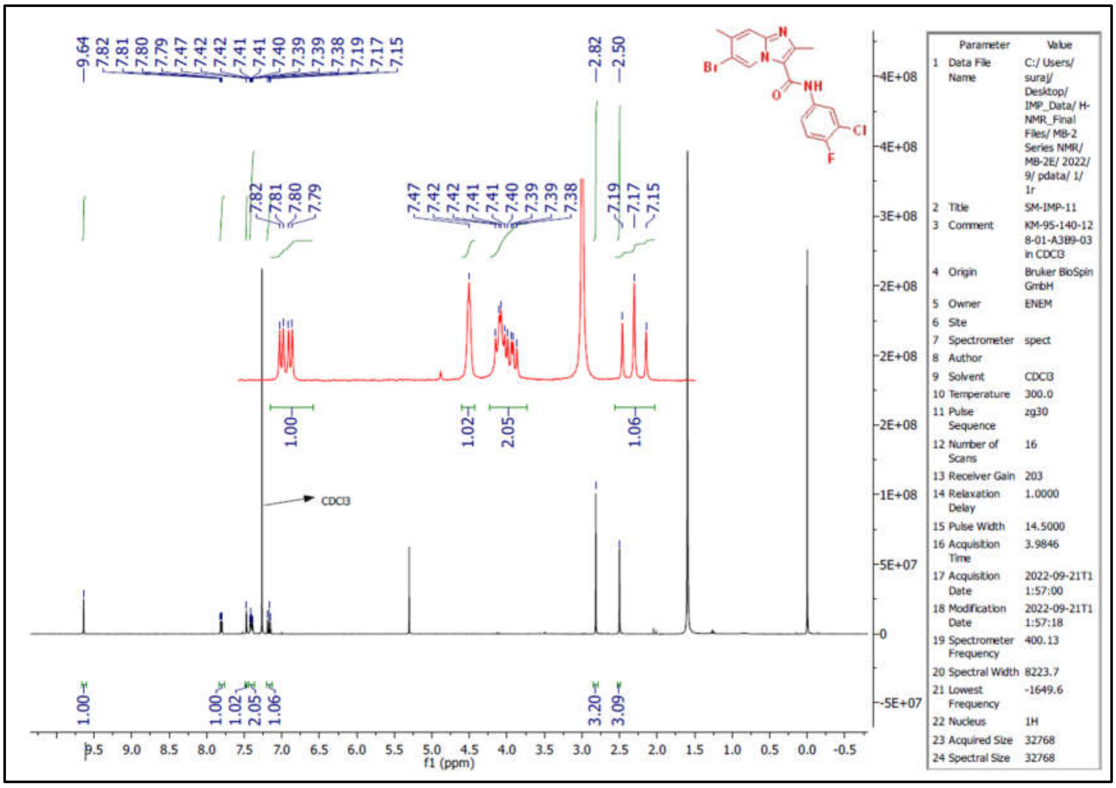

Figure S11. ^{1}H-NMR spectra of 6-Bromo-N-(3-Chloro-4-Fluorophenyl)-2,7-Dimethylimidazo[1,2-a]Pyridine-3-Carboxamide (**SM-IMP-11**).

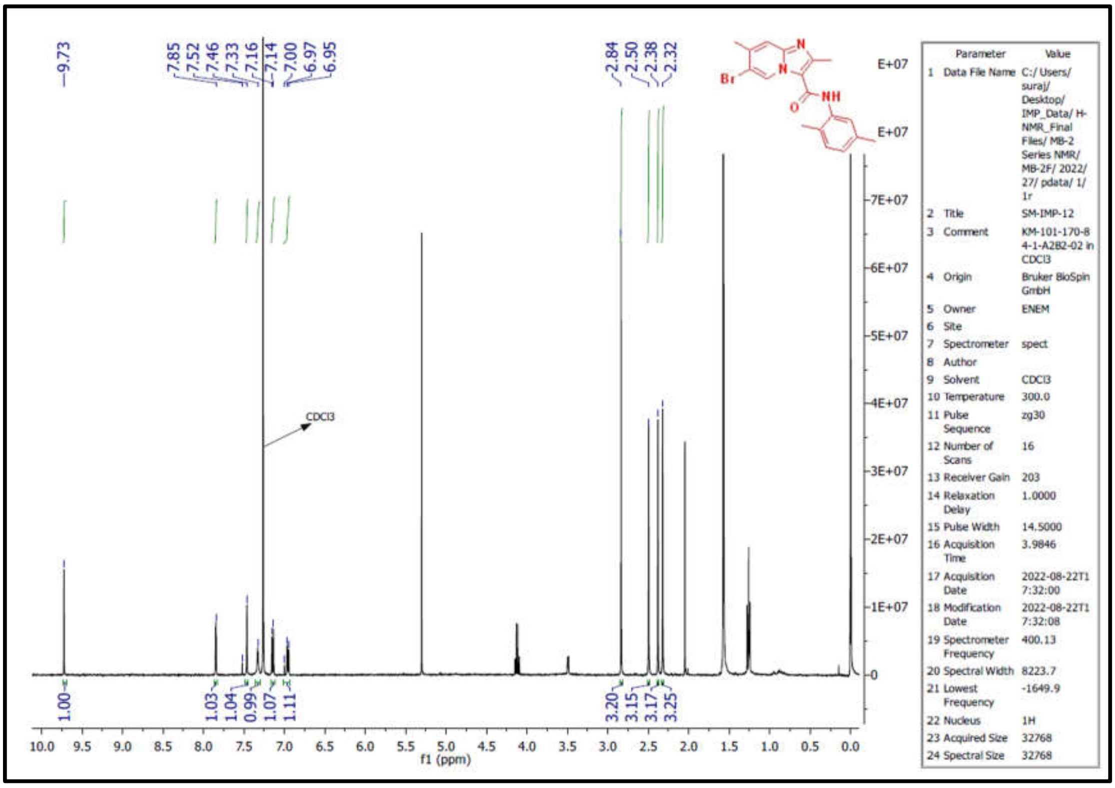

Figure S12. ^{1}H-NMR spectra of 6-Bromo-N-(2,5-Dimethylphenyl)-2,7-Dimethylimidazo[1,2-a]Pyridine-3-Carboxamide (**SM-IMP-12**).

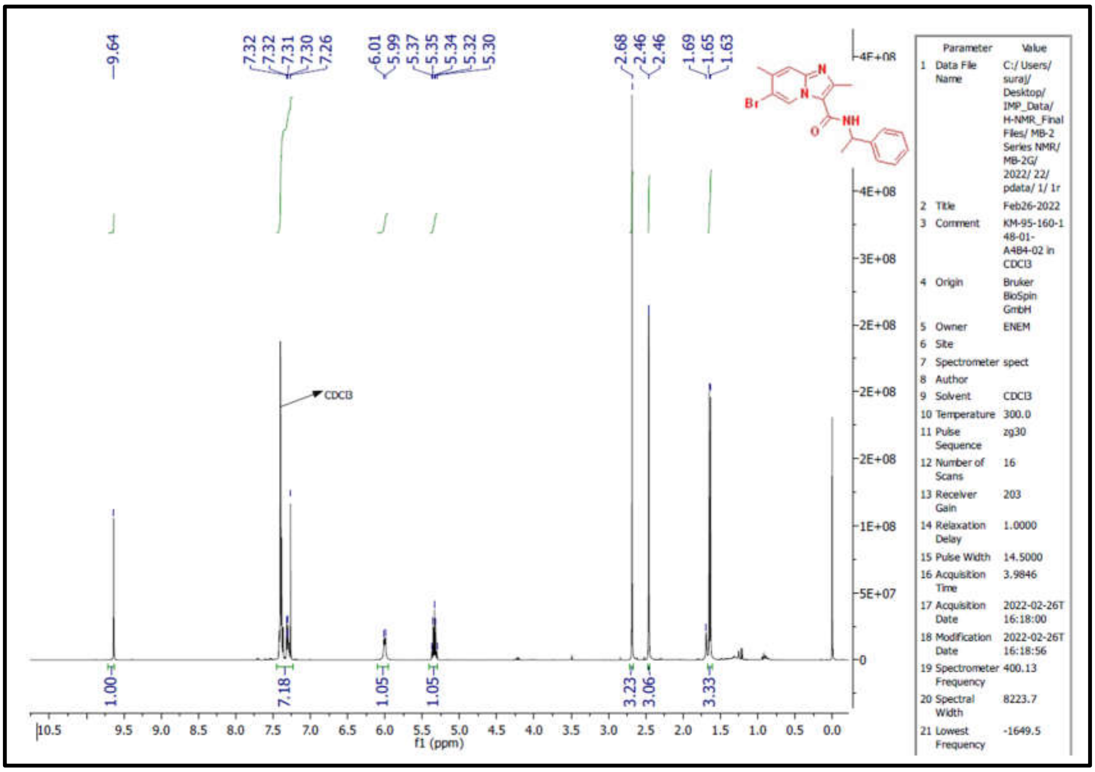

Figure S13. ^{1}H-NMR spectra of 6-Bromo-2,7-Dimethyl-N-(1-Phenylethyl)Imidazo[1,2-a]Pyridine-3-Carboxamide (**SM-IMP-13**).

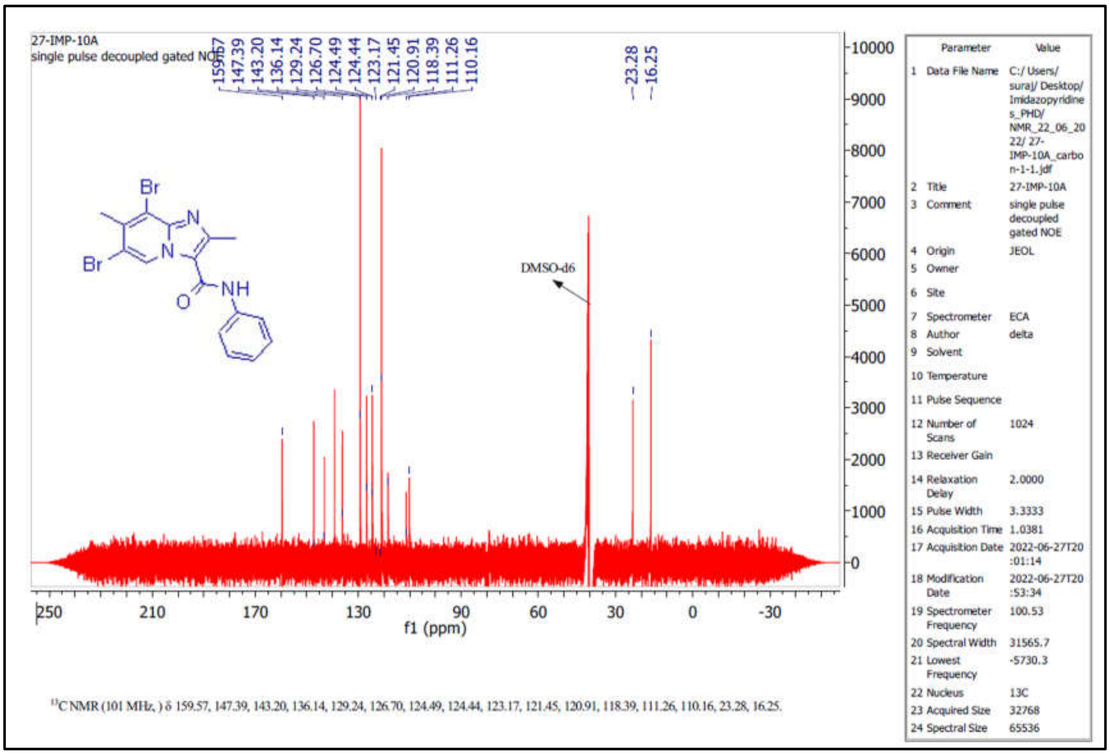

Figure S14. ^{13}C-NMR spectra of 6,8-dibromo-2,7-dimethyl-N-phenylimidazo[1,2-a]pyridine-3-carboxamide (**SM-IMP-01**).

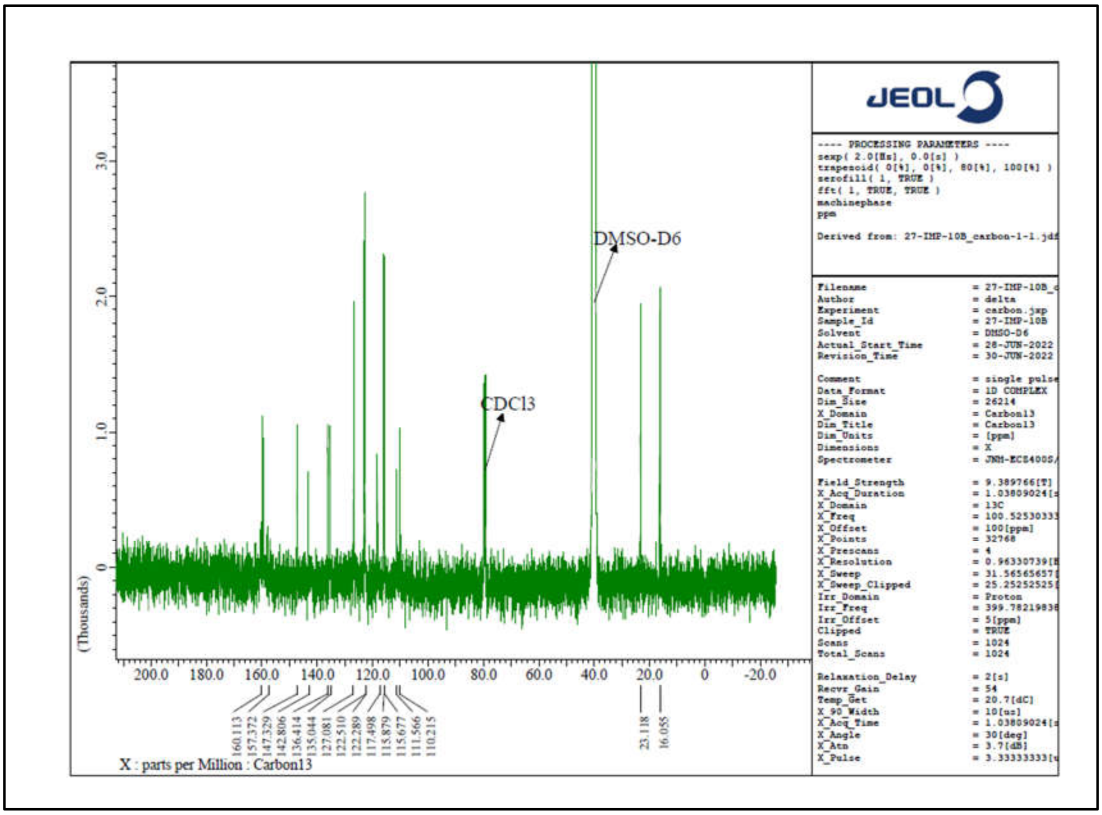

Figure S15. ^{13}C-NMR spectra of 6,8-Dibromo-2,7-Dimethyl-N-(4-Fluorophenyl)Imidazo[1,2-a]Pyridine-3-Carboxamide (**SM-IMP-02**).

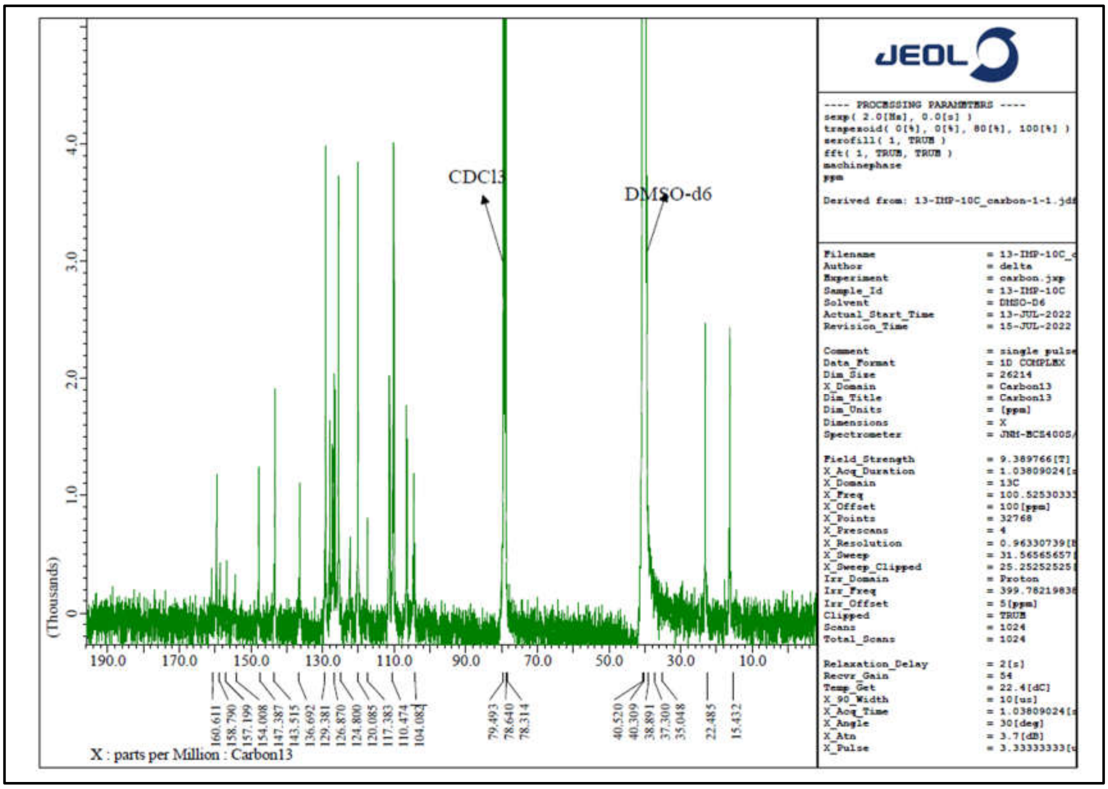

Figure S16. ^{13}C-NMR spectra of 6,8-Dibromo-N-(2,4-Difluorophenyl)-2,7-Dimethylimidazo[1,2-a]Pyridine-3-Carboxamide (**SM-IMP-03**).

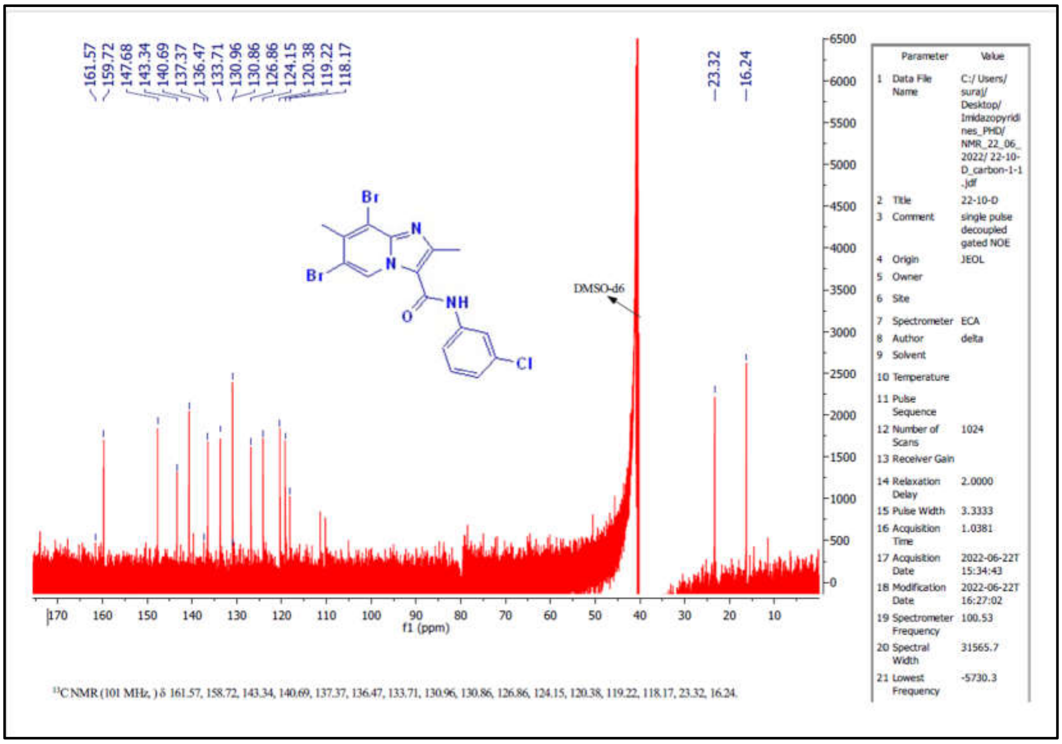

Figure S17. ^{13}C-NMR spectra of 6,8-Dibromo-N-(3-Chlorophenyl)-2,7-Dimethylimidazo[1,2-a]Pyridine-3-Carboxamide (**SM-IMP-04**).

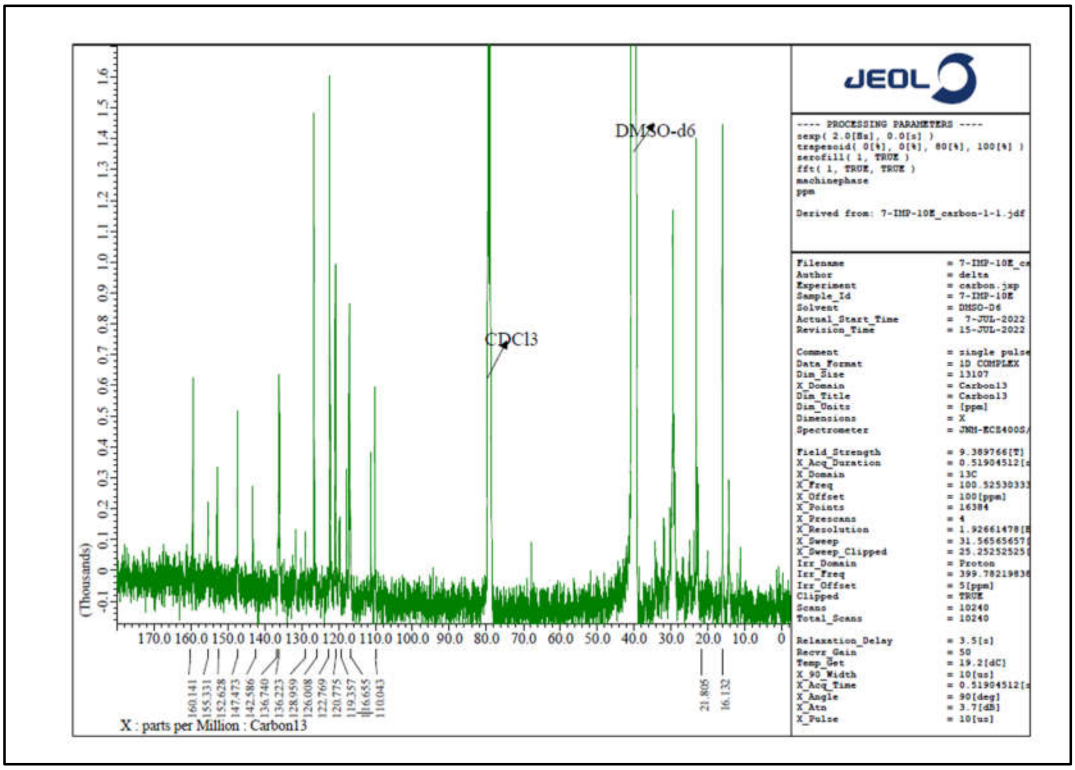

Figure S18. ^{13}C-NMR spectra of 6,8-Dibromo-N-(3-Chloro-4-Fluorophenyl)-2,7-Dimethylimidazo[1,2-a]Pyridine-3-Carboxamide (**SM-IMP-05**).

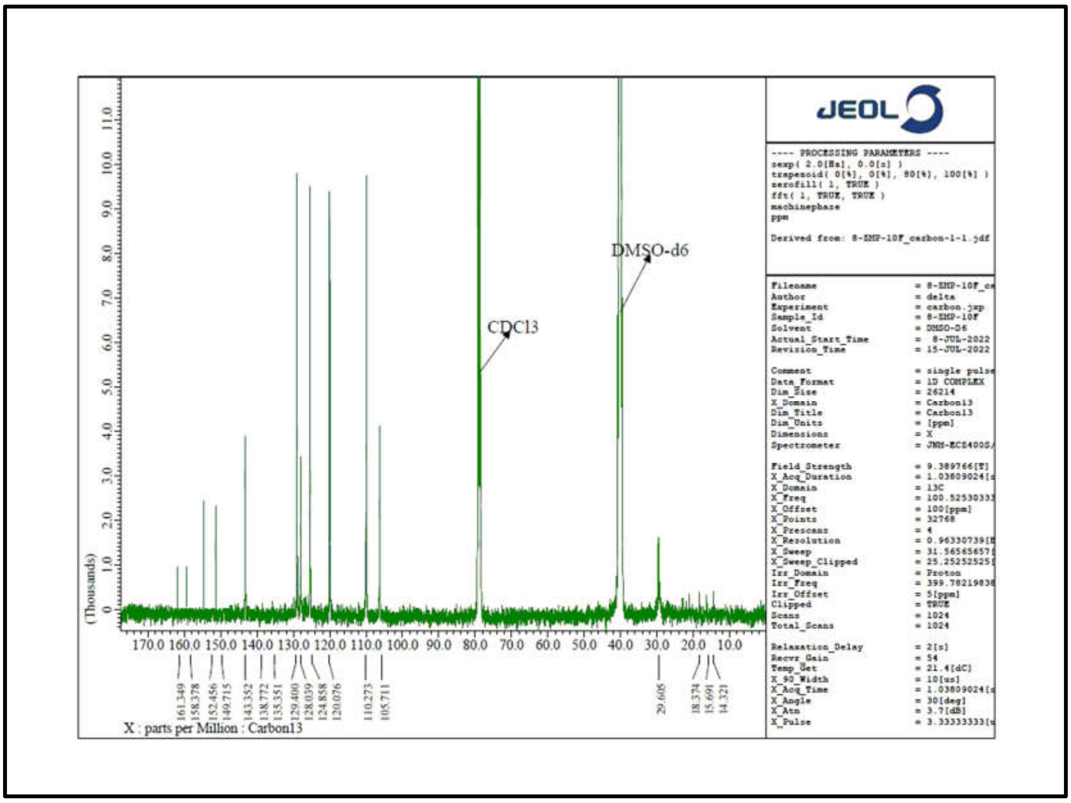

Figure S19. ^{13}C-NMR spectra of 6,8-Dibromo-N-(2,5-Dimethylphenyl)-2,7-Dimethylimidazo[1,2-a]Pyridine-3-Carboxamide (**SM-IMP-06**).

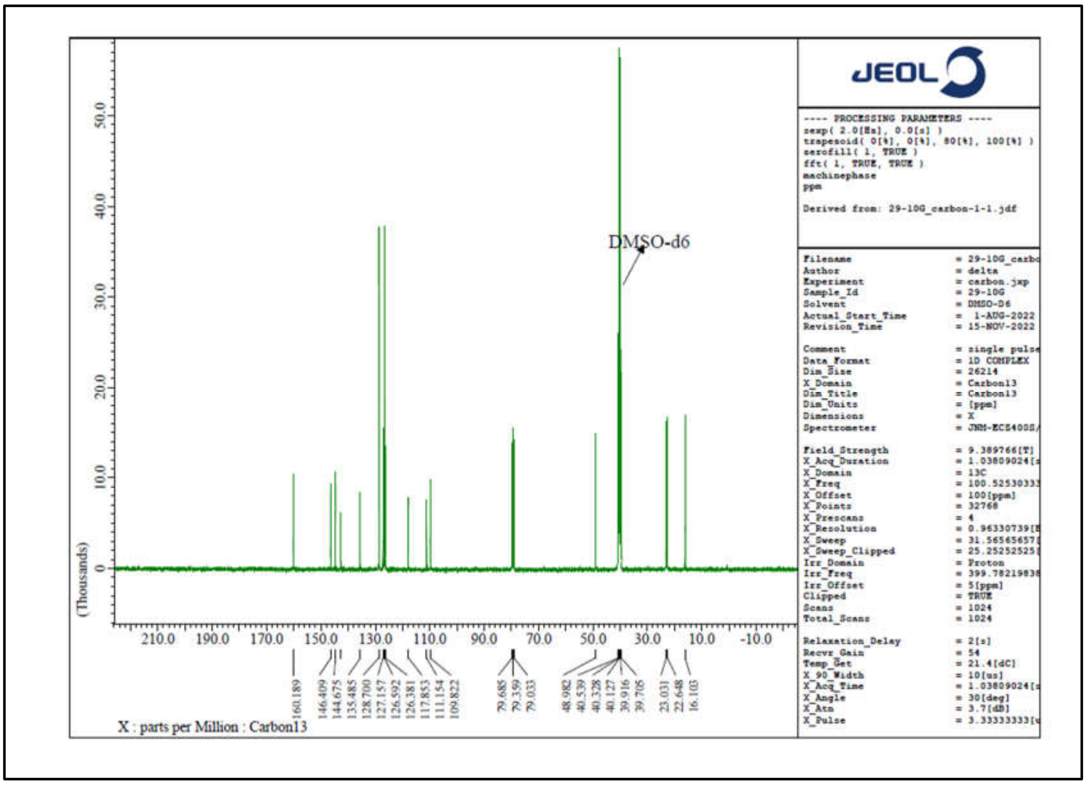

Figure S20. ^{13}C-NMR spectra of 6,8-Dibromo-2,7-Dimethyl-N-(1-Phenylethyl)Imidazo[1,2-a]Pyridine-3-Carboxamide (**SM-IMP-07**).

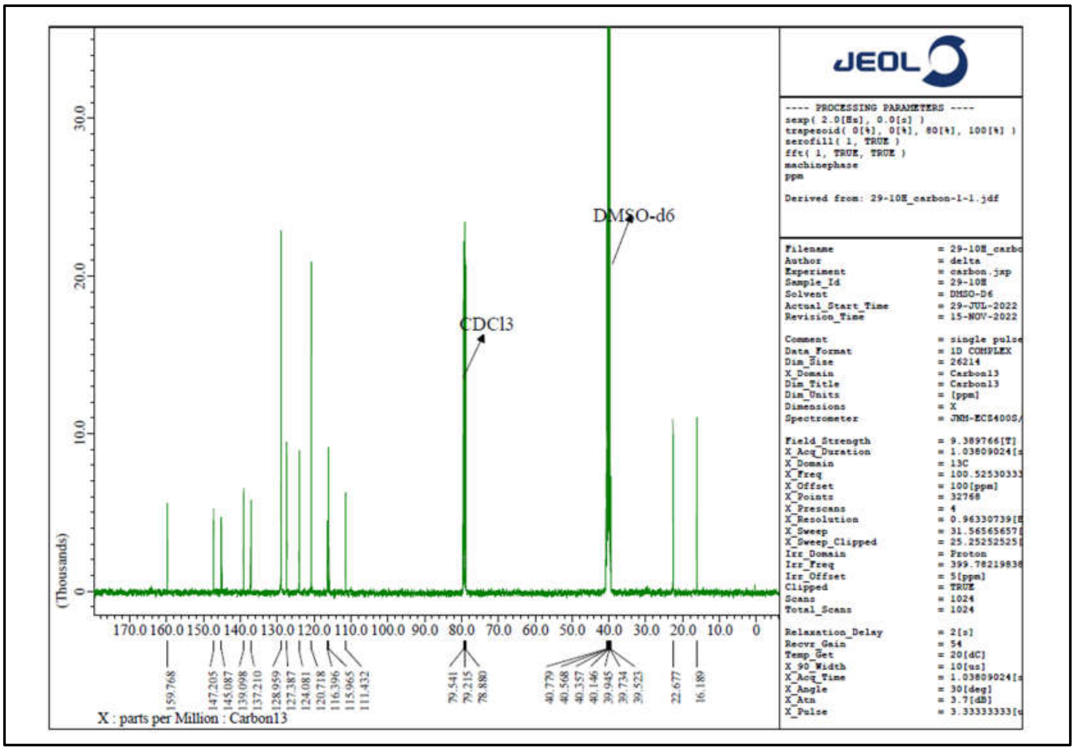

Figure S21. ^{13}C-NMR spectra of 6-Bromo-2,7-Dimethyl-N-Phenylimidazo[1,2-a]Pyridine-3-Carboxamide (**SM-IMP-08**).

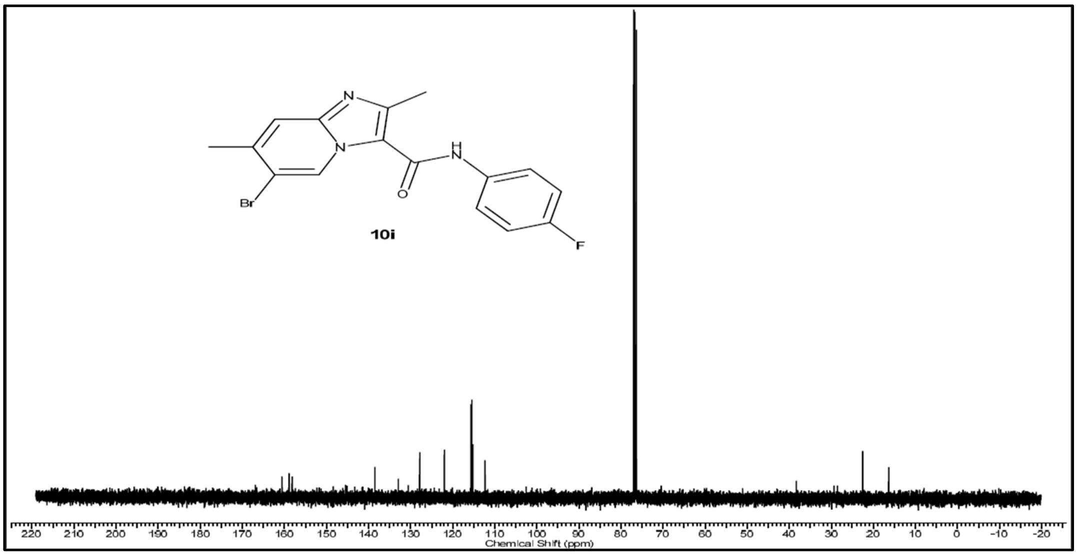

Figure S22. ^{13}C-NMR spectra of 6-Bromo-N-(4-Fluorophenyl)-2,7-Dimethyl Imidazo[1,2-a]Pyridine-3-Carboxamide (**SM-IMP-09**).

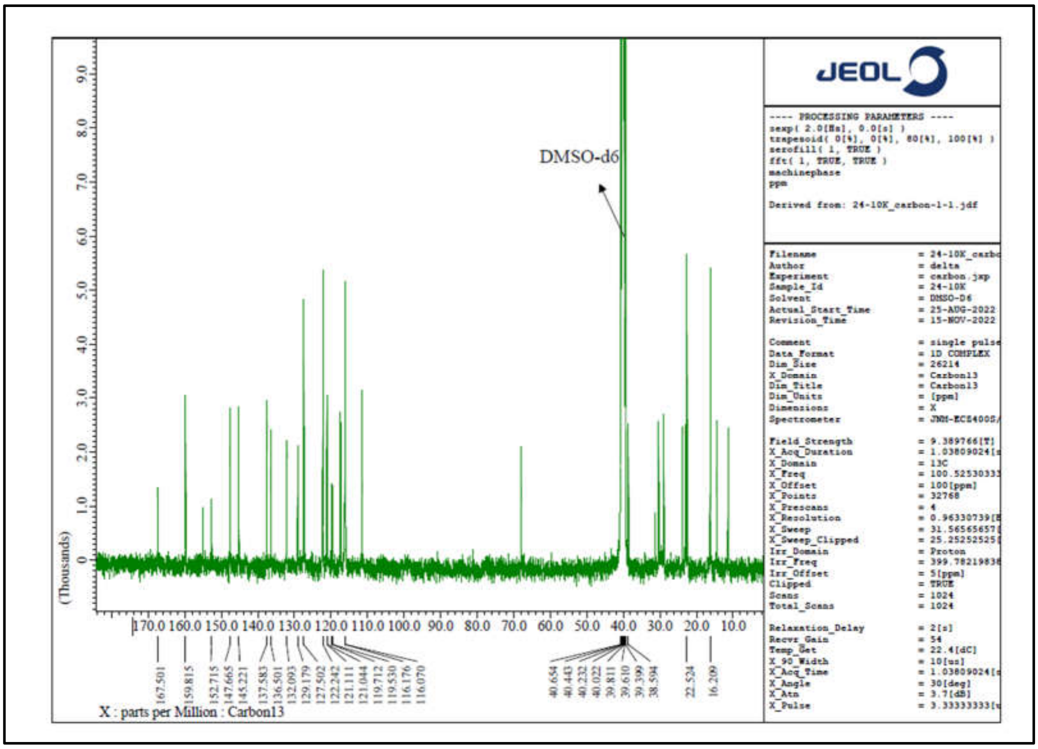

Figure S23. ^{13}C-NMR spectra of 6-Bromo-N-(3-Chloro-4-Fluorophenyl)-2,7-Dimethylimidazo[1,2-a]Pyridine-3-Carboxamide (**SM-IMP-11**).

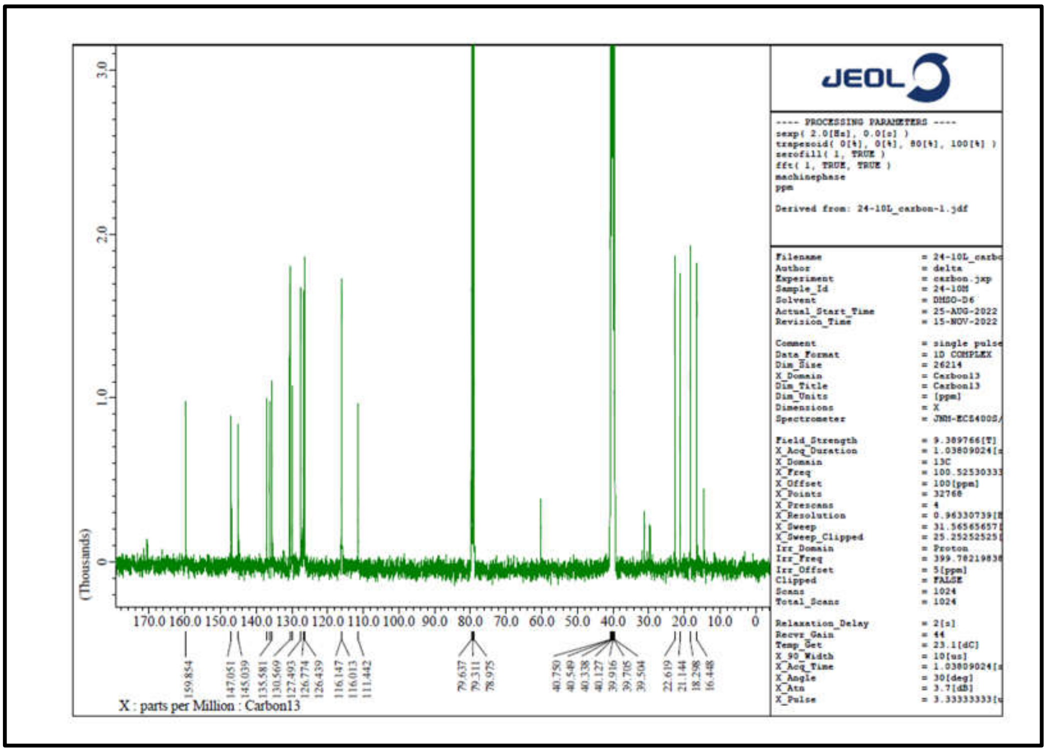

Figure S24. ^{13}C-NMR spectra of 6-Bromo-N-(2,5-Dimethylphenyl)-2,7-Dimethylimidazo[1,2-a]Pyridine-3-Carboxamide (**SM-IMP-12**).

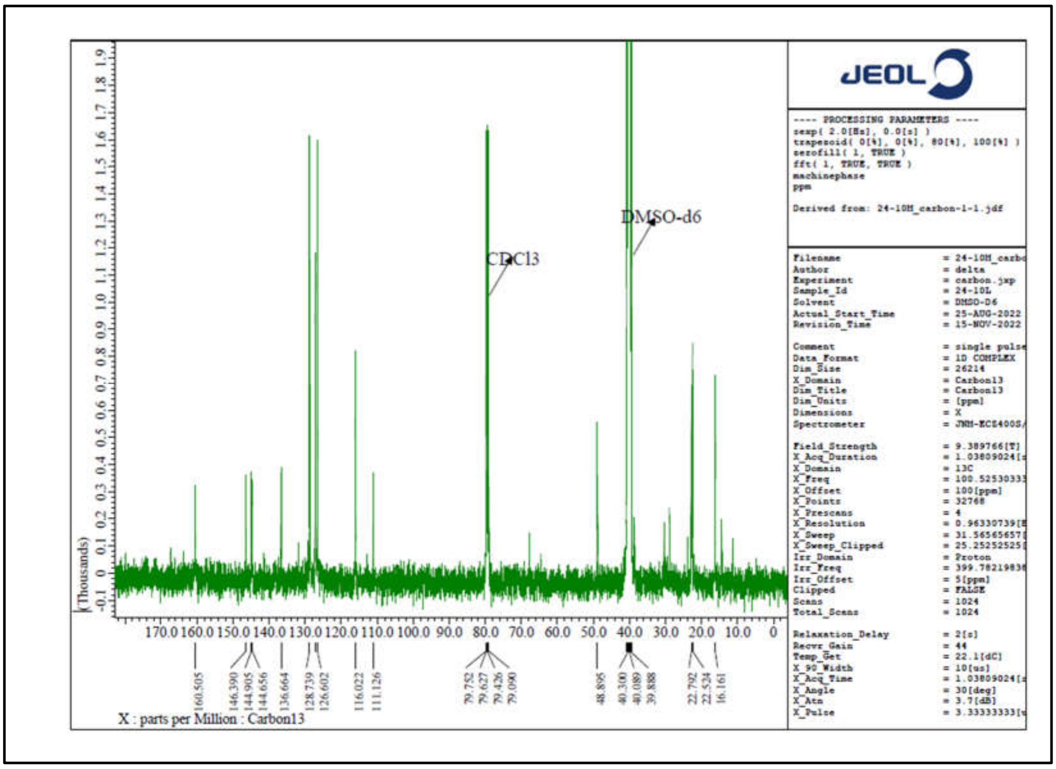

Figure S25. ^{13}C-NMR spectra of 6-Bromo-2,7-Dimethyl-N-(1-Phenylethyl)Imidazo[1,2-a]Pyridine-3-Carboxamide (**SM-IMP-13**).

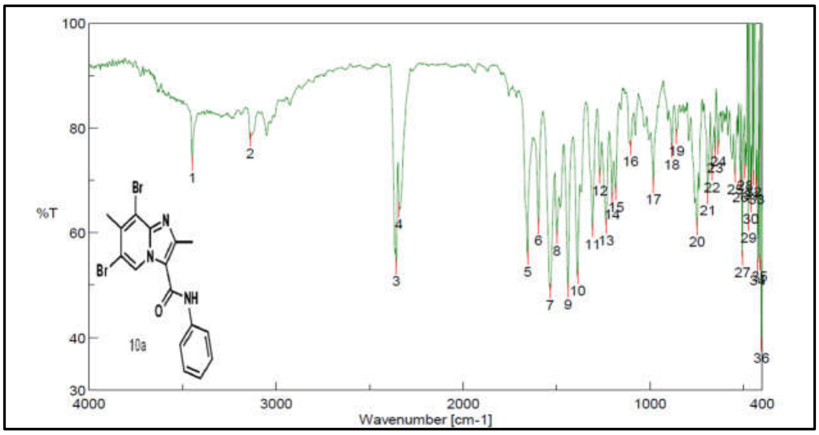

Figure S26. FT-IR spectra of 6,8-dibromo-2,7-dimethyl-N-phenylimidazo[1,2-a]pyridine-3-carboxamide (**SM-IMP-01**).

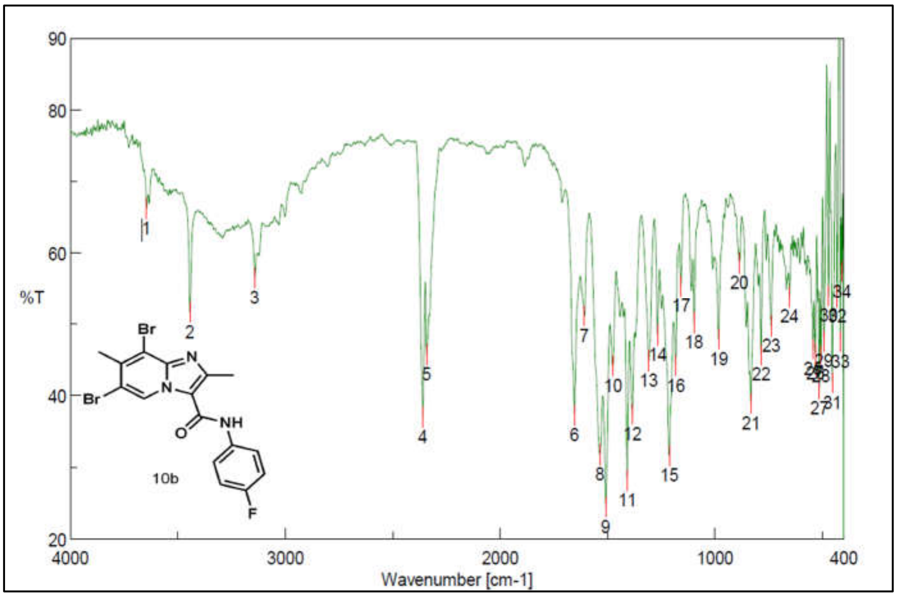

Figure S27. FT-IR spectra of 6,8-Dibromo-2,7-Dimethyl-N-(4-Fluorophenyl)Imidazo[1,2-a]Pyridine-3-Carboxamide (**SM-IMP-02**). .

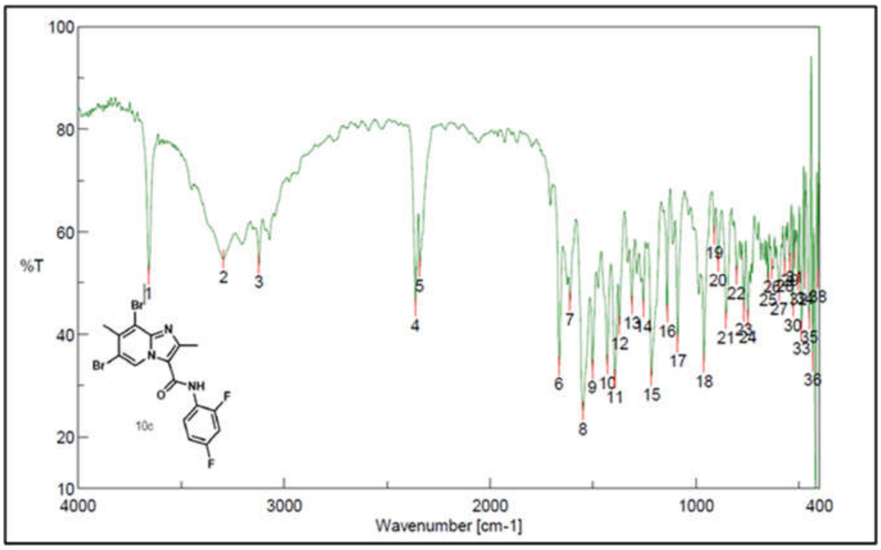

Figure S28. FT-IR spectra of 6,8-Dibromo-N-(2,4-Difluorophenyl)-2,7-Dimethylimidazo[1,2-a]Pyridine-3-Carboxamide (**SM-IMP-03**).

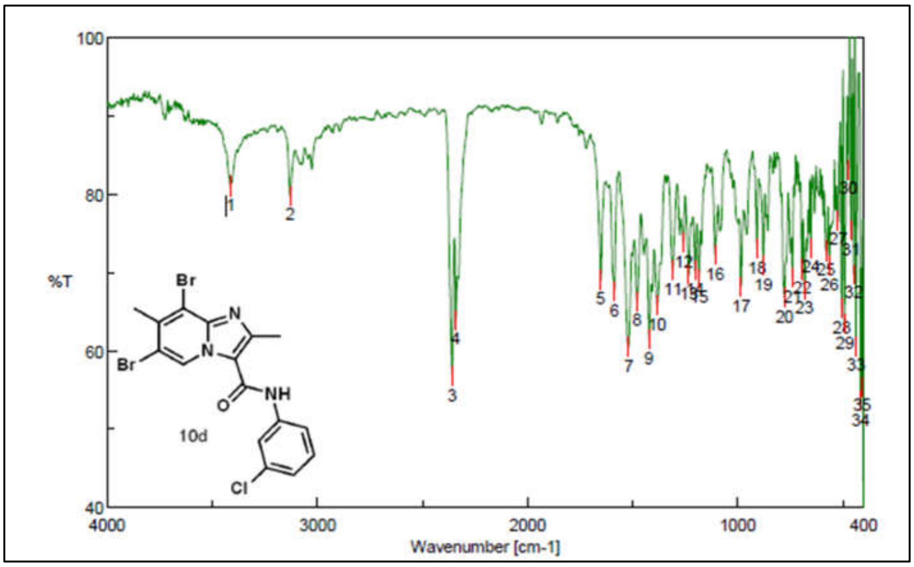

Figure S29. FT-IR spectra of 6,8-Dibromo-N-(3-Chlorophenyl)-2,7-Dimethylimidazo[1,2-a]Pyridine-3-Carboxamide (**SM-IMP-04**).

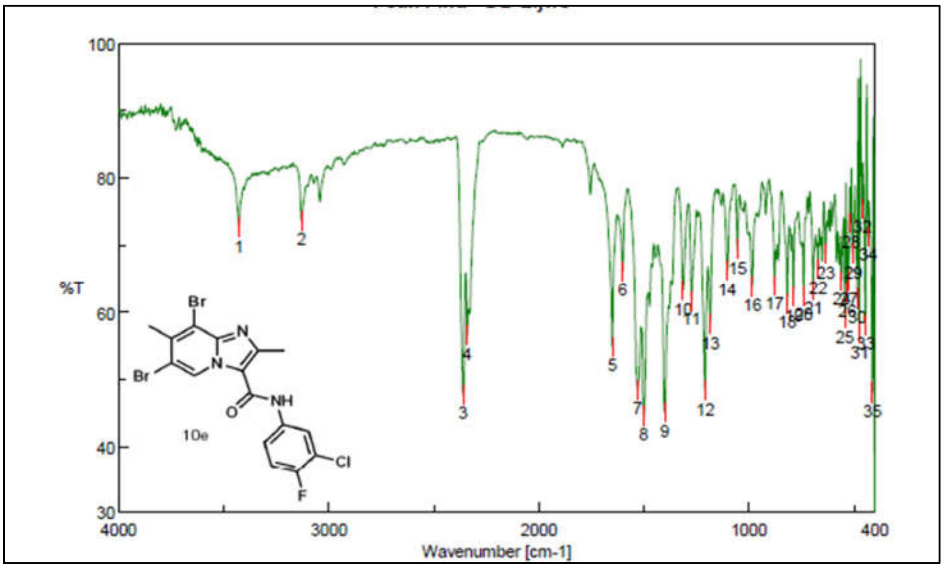

Figure S30. FT-IR spectra of 6,8-Dibromo-N-(3-Chloro-4-Fluorophenyl)-2,7-Dimethylimidazo[1,2-a]Pyridine-3-Carboxamide (**SM-IMP-05**).

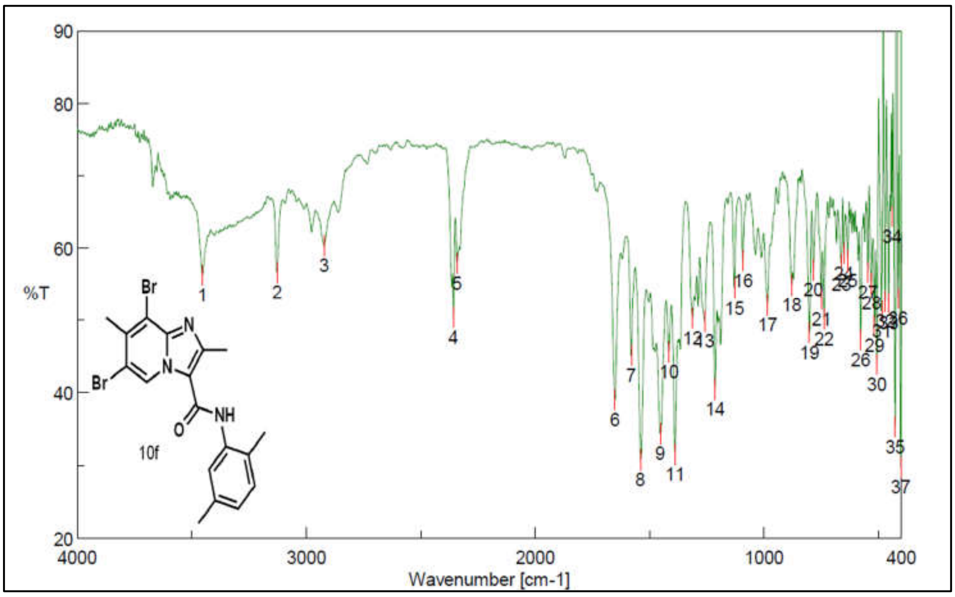

Figure S31. FT-IR spectra of 6,8-Dibromo-N-(2,5-Dimethylphenyl)-2,7-Dimethylimidazo[1,2-a]Pyridine-3-Carboxamide (**SM-IMP-06**).

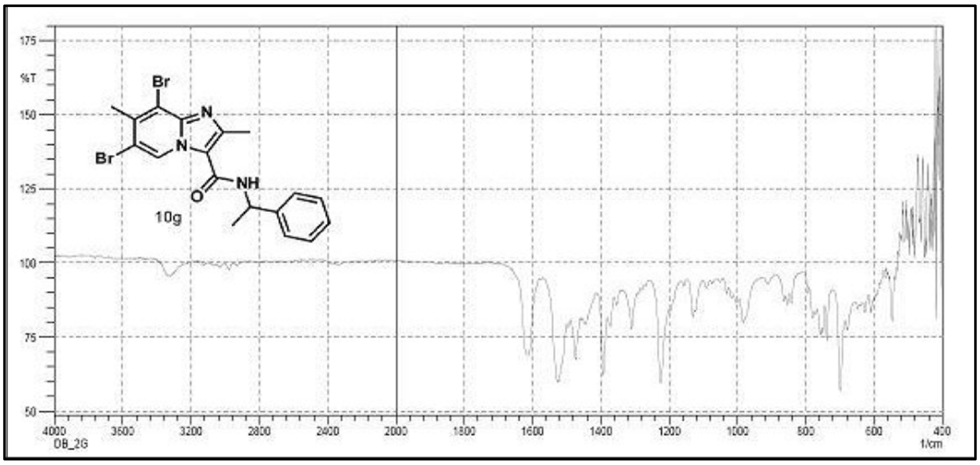

Figure S32. FT-IR spectra of 6,8-Dibromo-2,7-Dimethyl-N-(1-Phenylethyl)Imidazo[1,2-a]Pyridine-3-Carboxamide (**SM-IMP-07**).

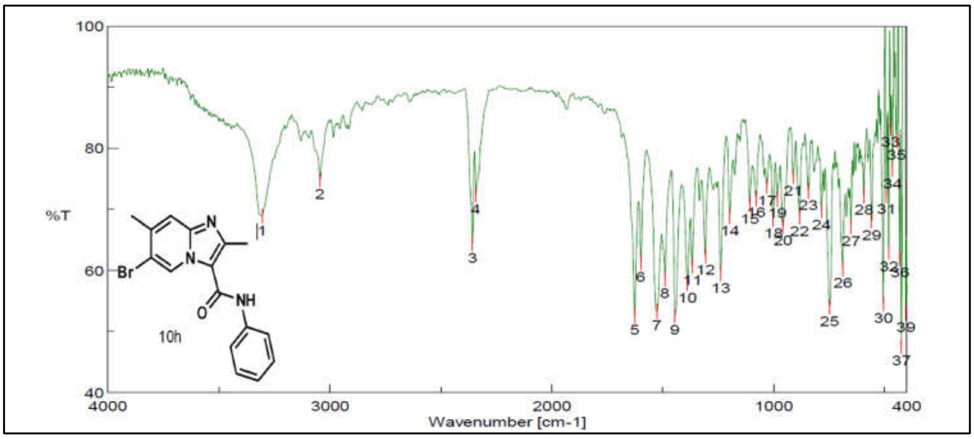

Figure S33. FT-IR spectra of 6-Bromo-2,7-Dimethyl-N-Phenylimidazo[1,2-a]Pyridine-3-Carboxamide (**SM-IMP-08**).

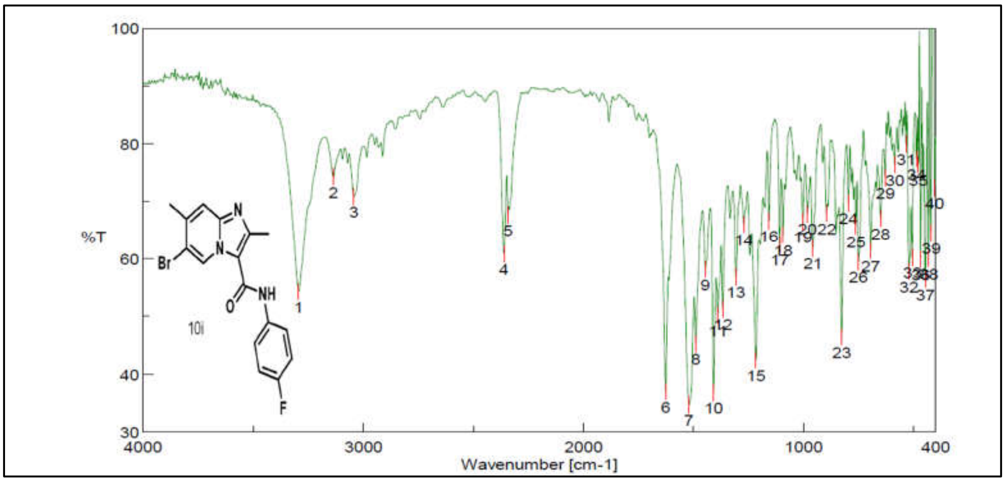

Figure S34. FT-IR spectra of 6-Bromo-N-(4-Fluorophenyl)-2,7-Dimethyl Imidazo[1,2-a]Pyridine-3-Carboxamide (**SM-IMP-09**).

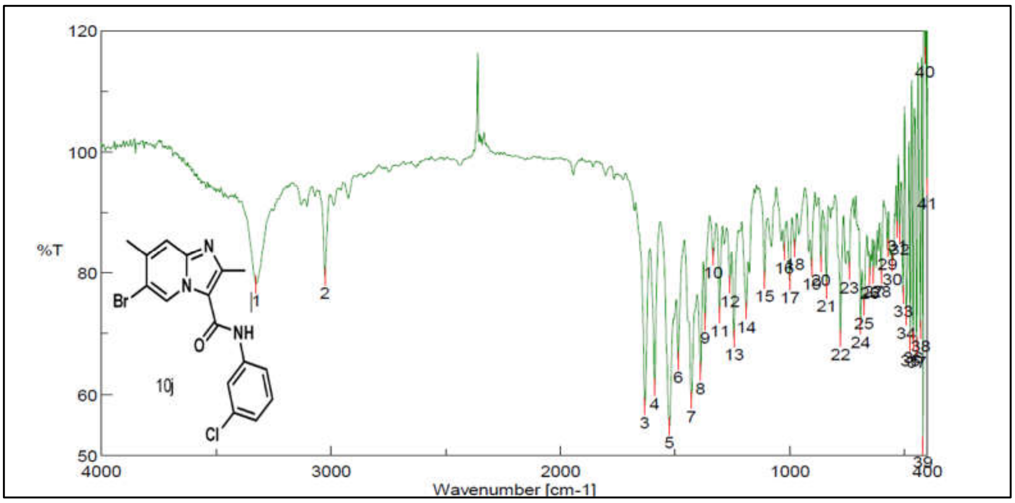

Figure S35. FT-IR spectra of 6-Bromo-N-(3-Chlorophenyl)-2,7-Dimethyl Imidazo[1,2-a]Pyridine-3-Carboxamide (**SM-IMP-10**).

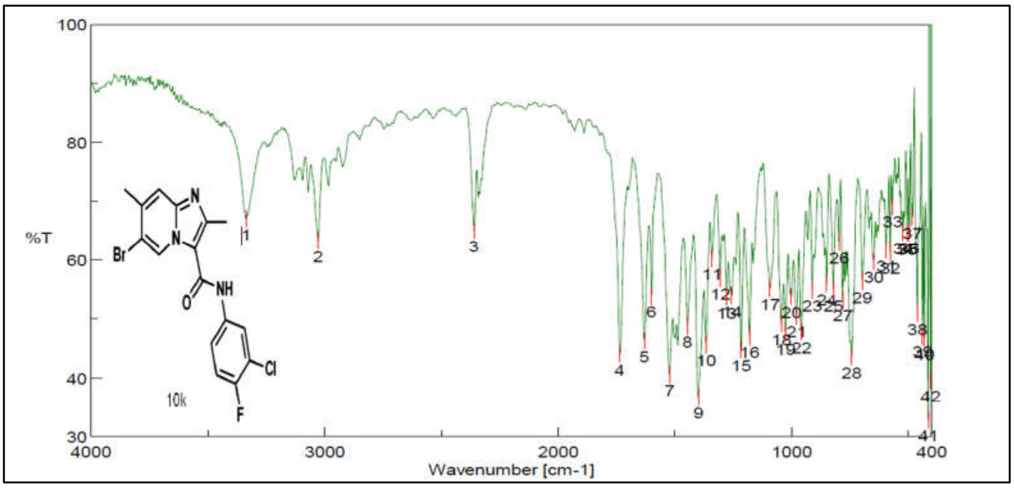

Figure S36. FT-IR spectra of 6-Bromo-N-(3-Chloro-4-Fluorophenyl)-2,7-Dimethylimidazo[1,2-a]Pyridine-3-Carboxamide (**SM-IMP-11**).

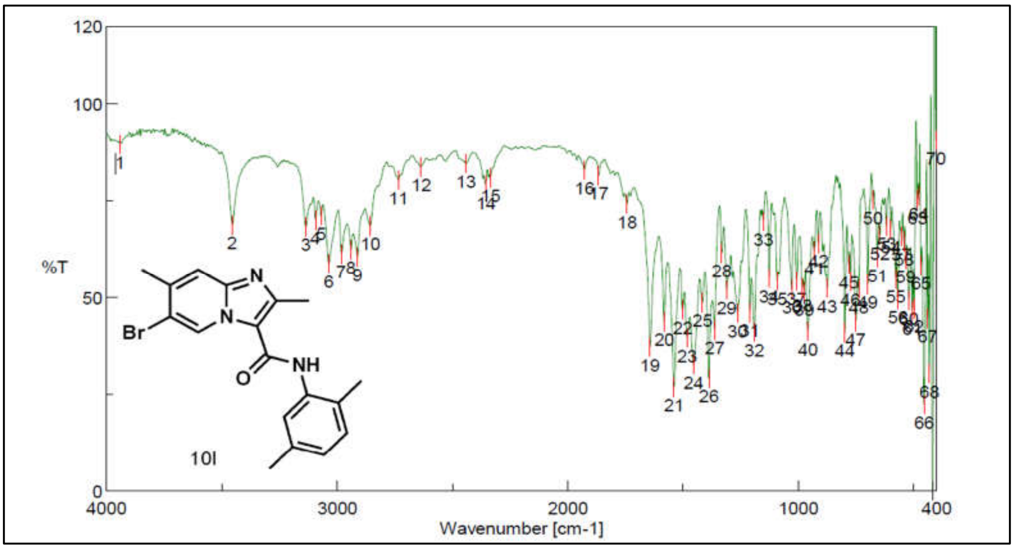

Figure S37. FT-IR spectra of 6-Bromo-N-(2,5-Dimethylphenyl)-2,7-Dimethylimidazo[1,2-a]Pyridine-3-Carboxamide (**SM-IMP-12**).

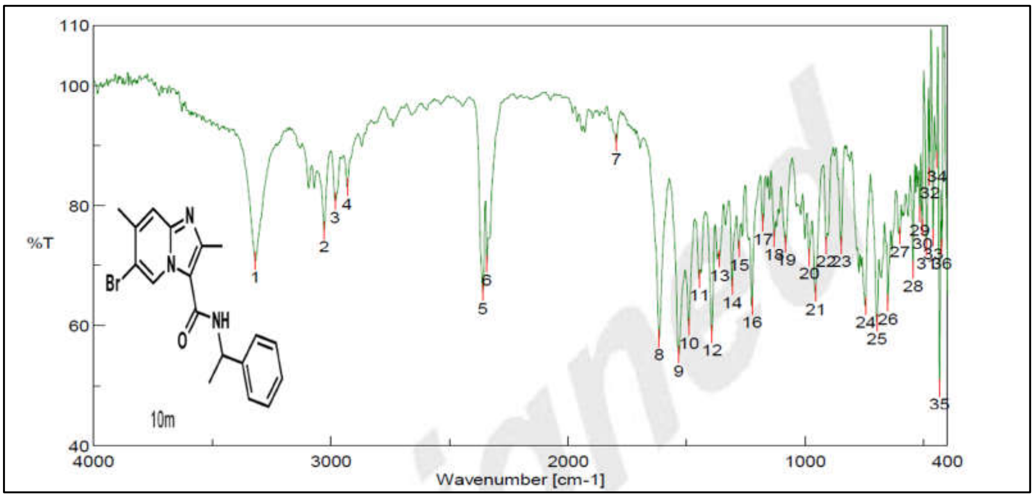

Figure S38. FT-IR spectra of 6-Bromo-2,7-Dimethyl-N-(1-Phenylethyl)Imidazo[1,2-a]Pyridine-3-Carboxamide (**SM-IMP-13**).

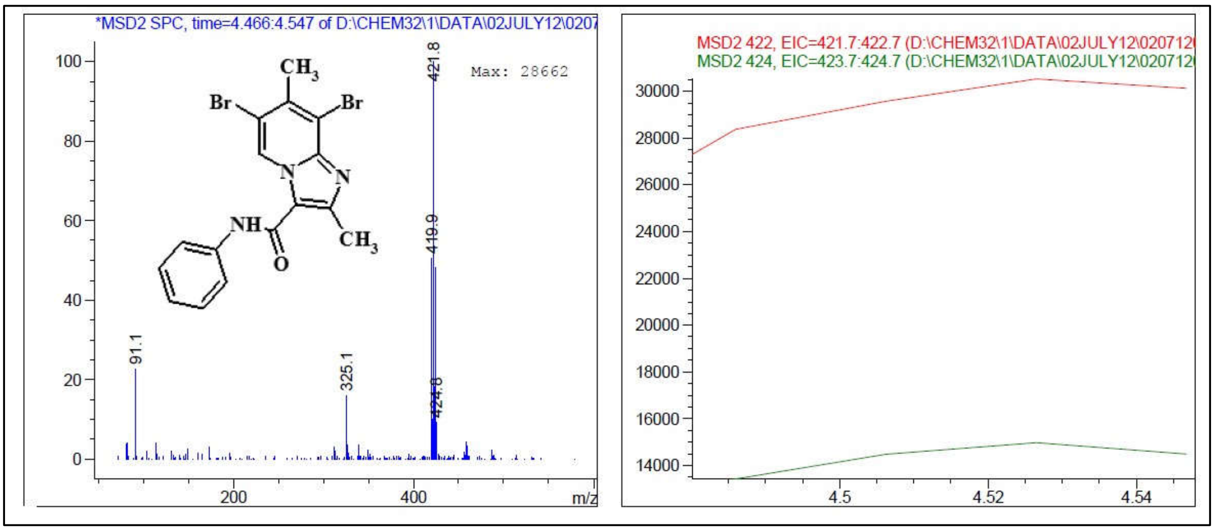

Figure S39. Mass spectra of 6,8-dibromo-2,7-dimethyl-N-phenylimidazo[1,2-a]pyridine-3-carboxamide (**SM-IMP-01**).

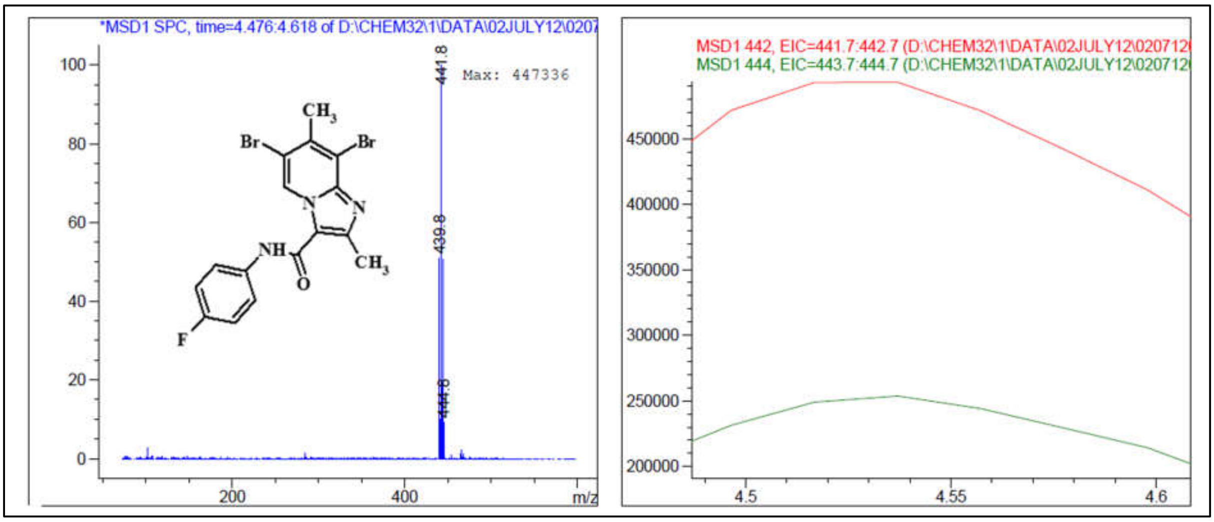

Figure S40. Mass spectra of 6,8-Dibromo-2,7-Dimethyl-N-(4-Fluorophenyl)Imidazo[1,2-a]Pyridine-3-Carboxamide (**SM-IMP-02**).

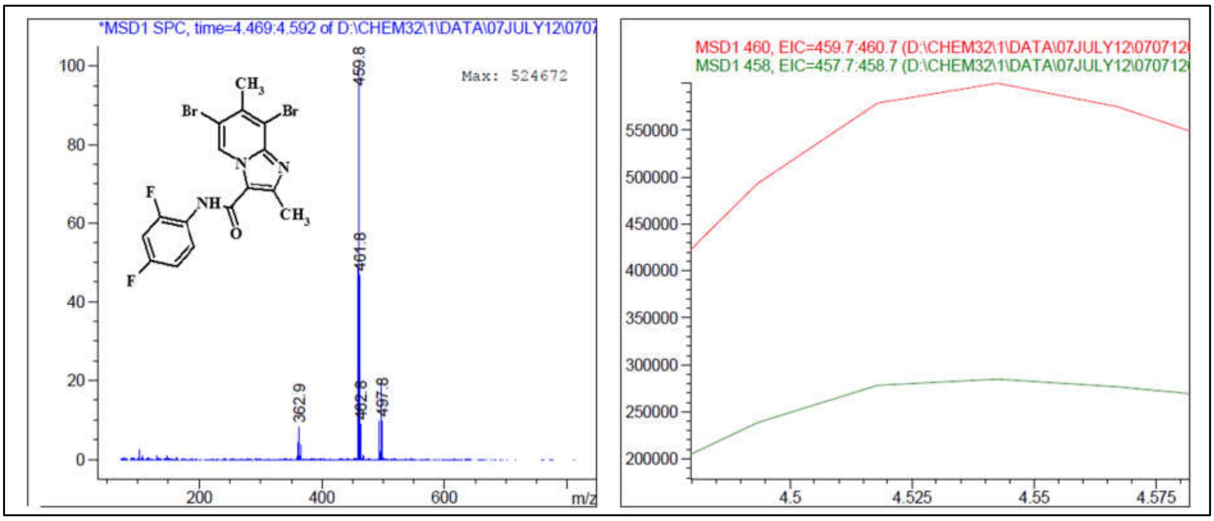

Figure S41. Mass spectra of 6,8-Dibromo-N-(2,4-Difluorophenyl)-2,7-Dimethylimidazo[1,2-a]Pyridine-3-Carboxamide (**SM-IMP-03**).

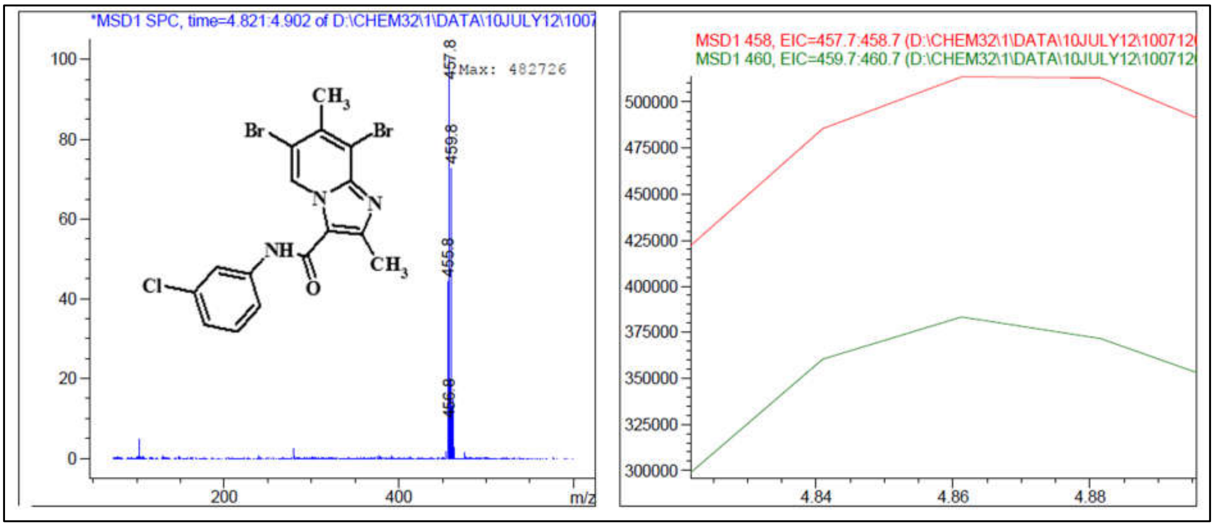

Figure S42. Mass spectra of 6,8-Dibromo-N-(3-Chlorophenyl)-2,7-Dimethylimidazo[1,2-a]Pyridine-3-Carboxamide (**SM-IMP-04**).

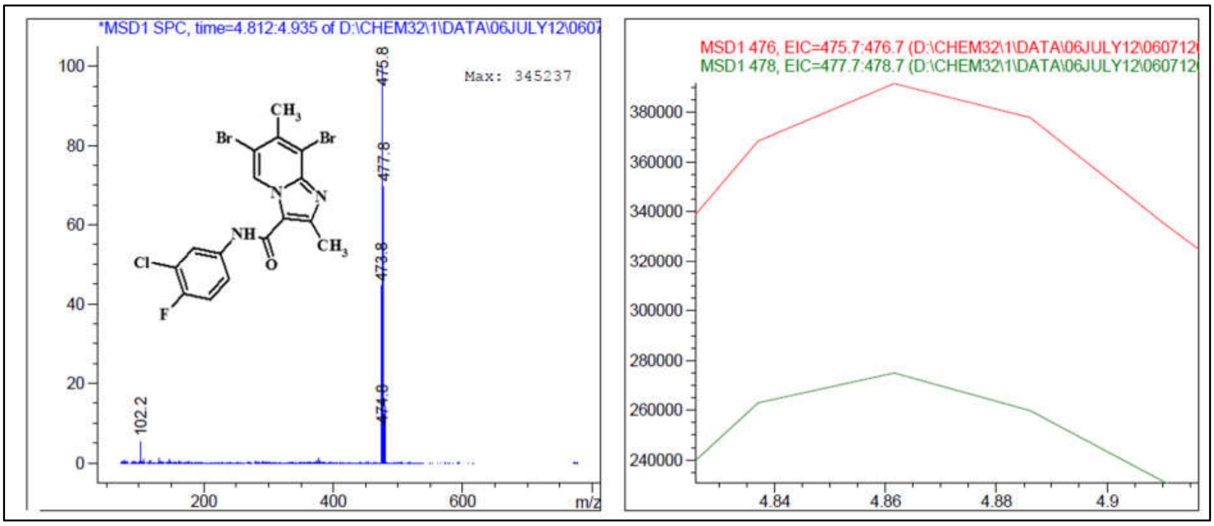

Figure S43. Mass spectra of 6,8-Dibromo-N-(3-Chloro-4-Fluorophenyl)-2,7-Dimethylimidazo[1,2-a]Pyridine-3-Carboxamide (**SM-IMP-05**).

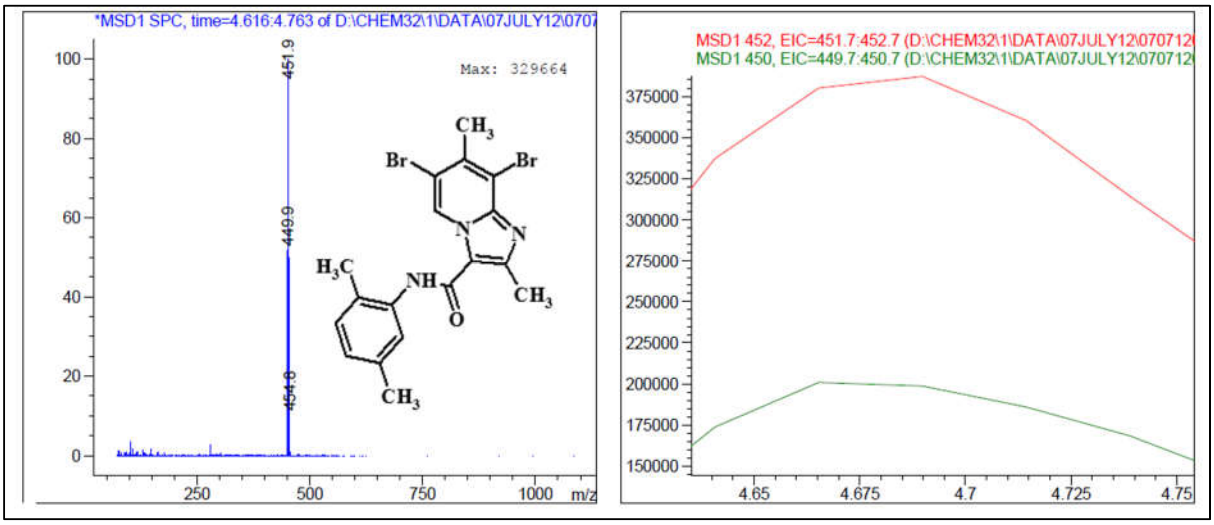

Figure S44. Mass spectra of 6,8-Dibromo-N-(2,5-Dimethylphenyl)-2,7-Dimethylimidazo[1,2-a]Pyridine-3-Carboxamide (**SM-IMP-06**).

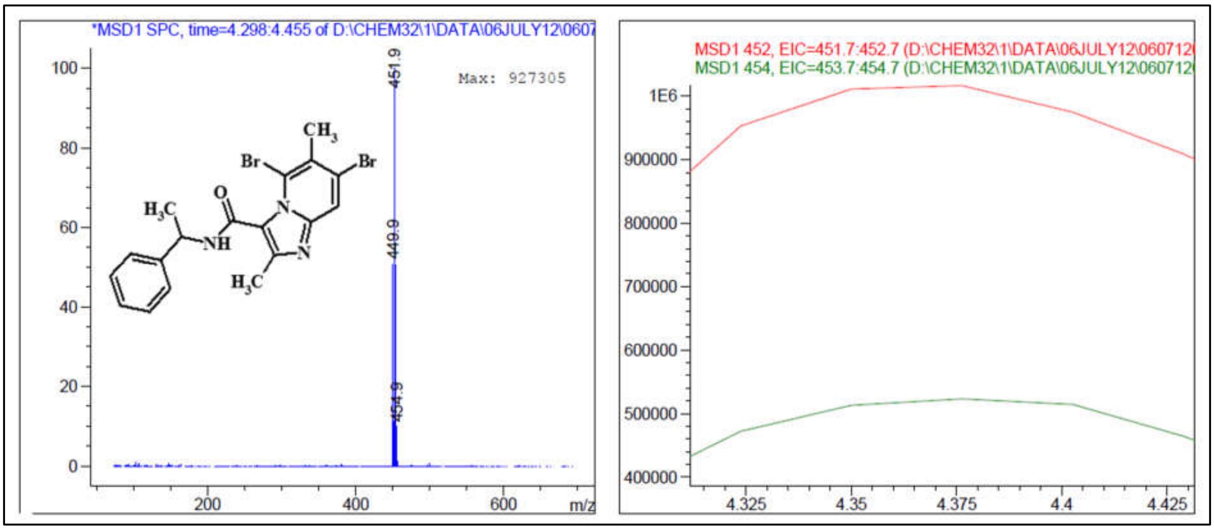

Figure S45. Mass spectra of 6,8-Dibromo-2,7-Dimethyl-N-(1-Phenylethyl)Imidazo[1,2-a]Pyridine-3-Carboxamide (**SM-IMP-07**).

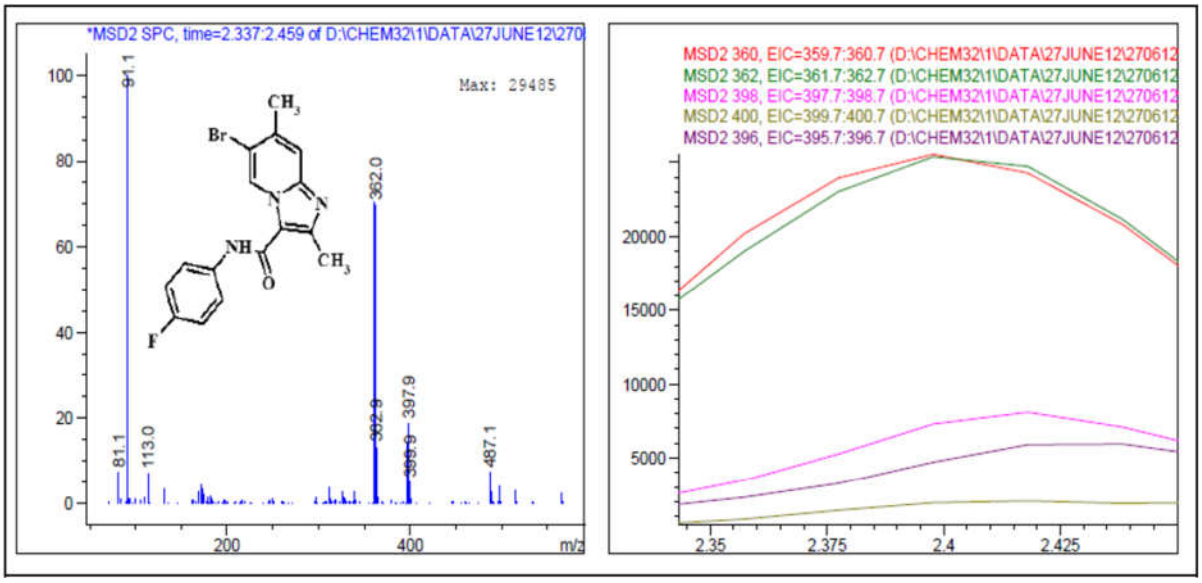

Figure S46. Mass spectra of 6-Bromo-N-(4-Fluorophenyl)-2,7-Dimethyl Imidazo[1,2-a]Pyridine-3-Carboxamide (**SM-IMP-09**).

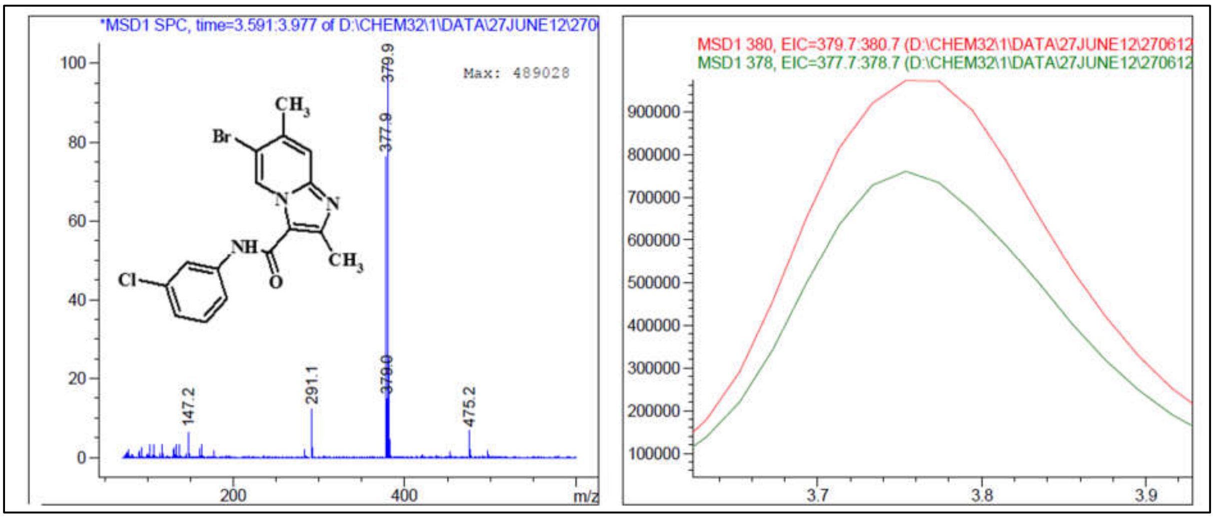

Figure S47. Mass spectra of 6-Bromo-N-(3-Chlorophenyl)-2,7-Dimethyl Imidazo[1,2-a]Pyridine-3-Carboxamide (**SM-IMP-10**).

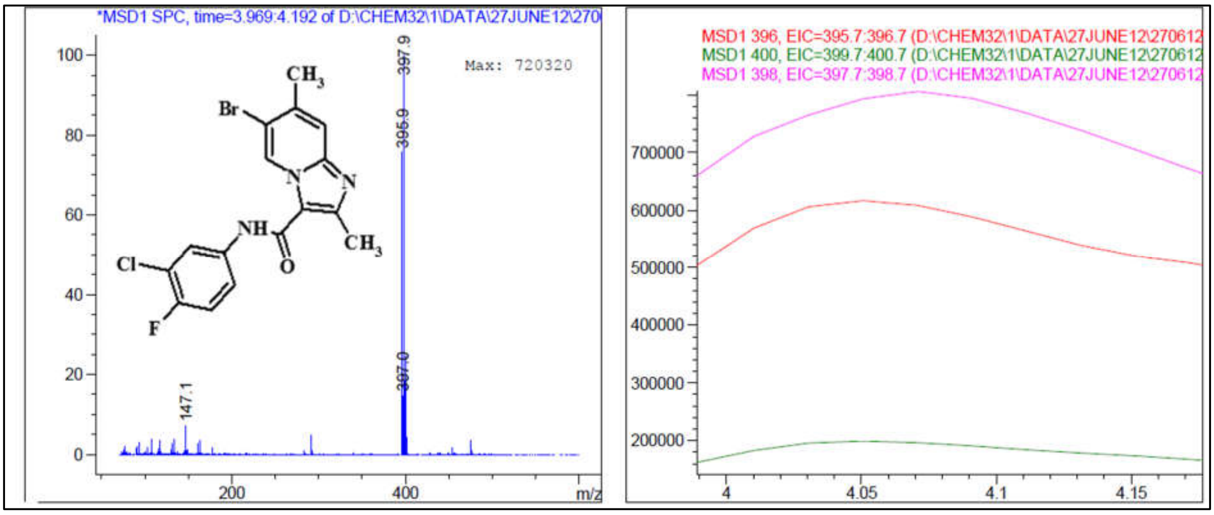

Figure S48. Mass spectra of 6-Bromo-N-(3-Chloro-4-Fluorophenyl)-2,7-Dimethylimidazo[1,2-a]Pyridine-3-Carboxamide (**SM-IMP-11**).

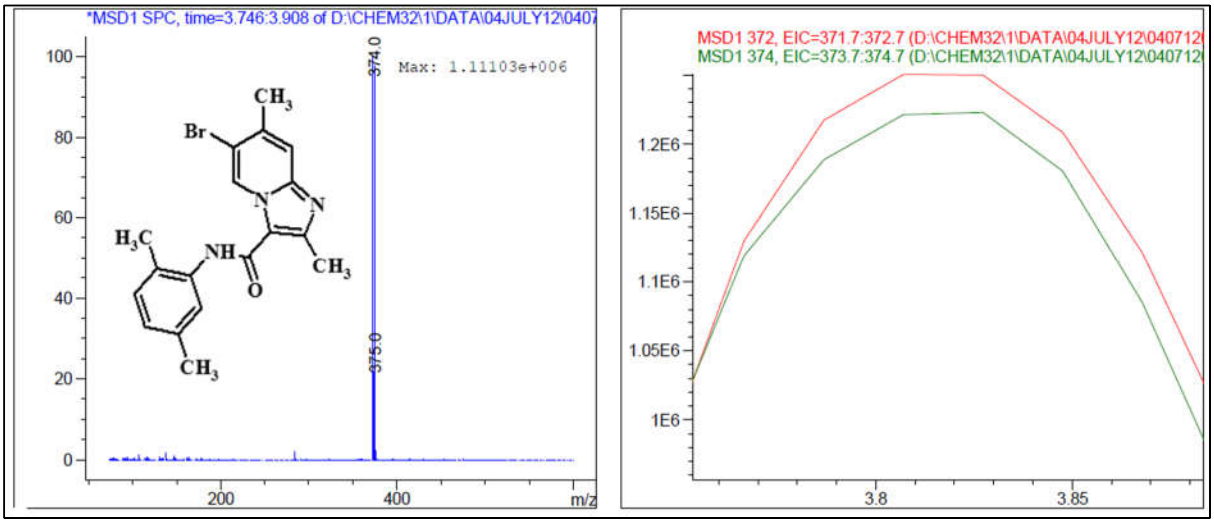

Figure S49. Mass spectra of 6-Bromo-N-(2,5-Dimethylphenyl)-2,7-Dimethylimidazo[1,2-a]Pyridine-3-Carboxamide (**SM-IMP-12**).

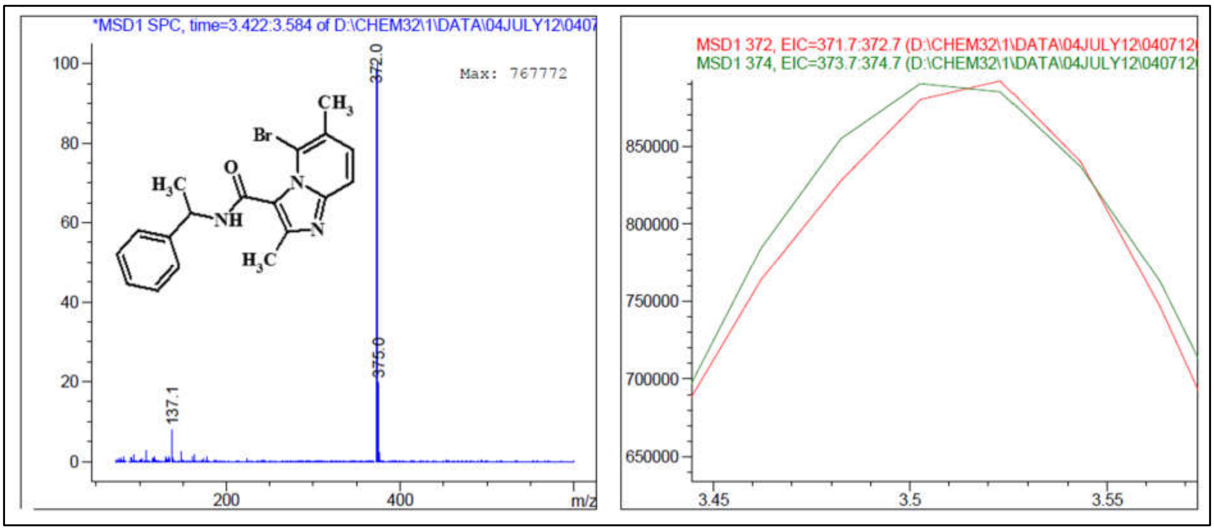

Figure S50. Mass spectra of 6-Bromo-2,7-Dimethyl-N-(1-Phenylethyl)Imidazo[1,2-a]Pyridine-3-Carboxamide (**SM-IMP-13**).

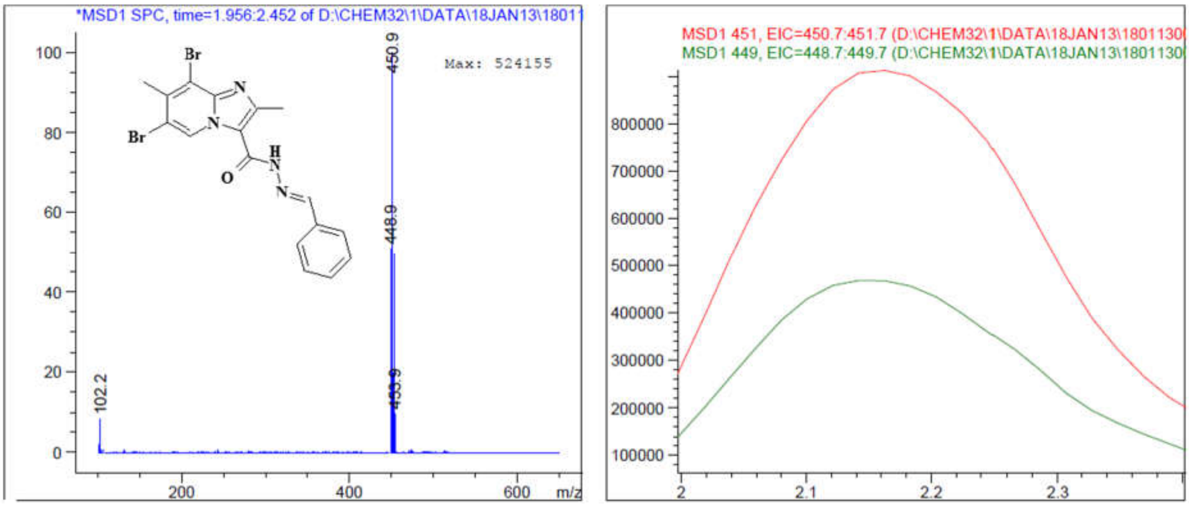

Figure S51. Mass spectra of (E)-N'-benzylidene-6,8-dibromo-2,7-dimethylimidazo[1,2-a]pyridine-3-carbohydrazide (**DA-01**).

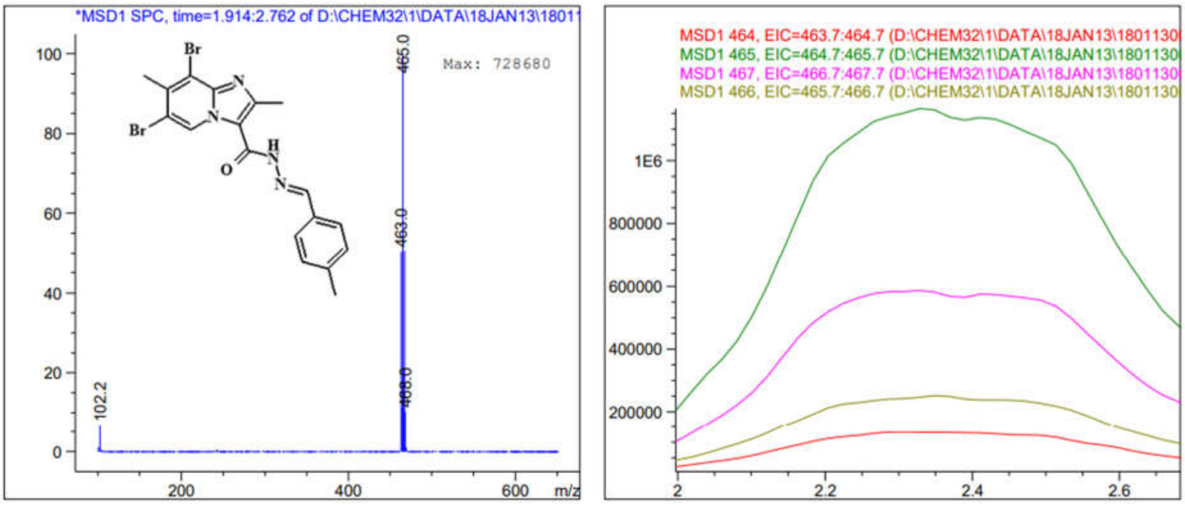

Figure S52. Mass spectra of (E)-6,8-dibromo-2,7-dimethyl-N'-(4-methylbenzylidene)imidazo[1,2-a]pyridine-3-carbohydrazide (**DA-02**).

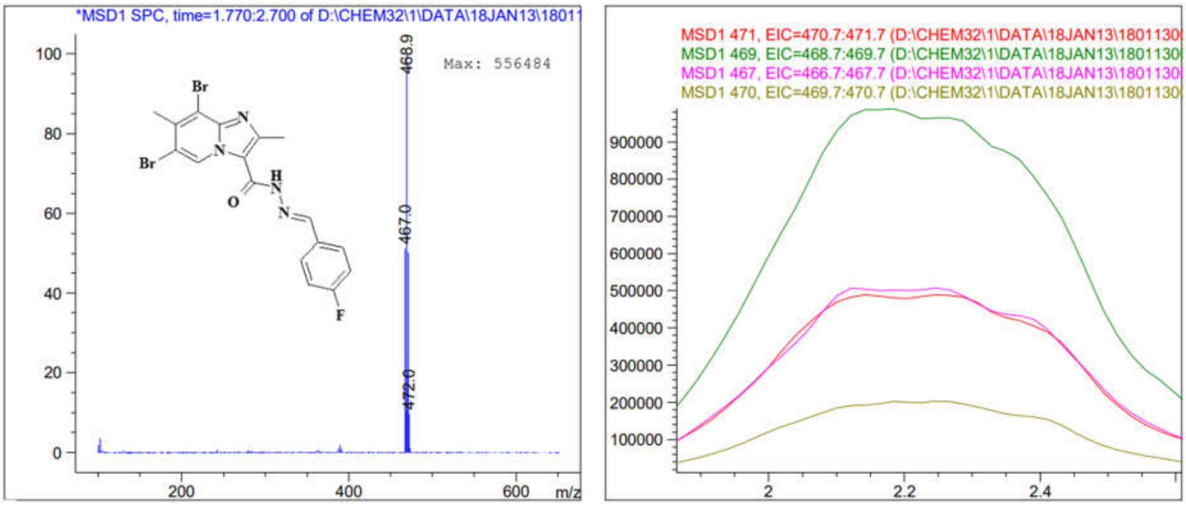

Figure S53. Mass spectra of (E)-6,8-dibromo-N'-(4-fluorobenzylidene)-2,7-dimethylimidazo[1,2-a]pyridine-3-carbohydrazide (**DA-03**).

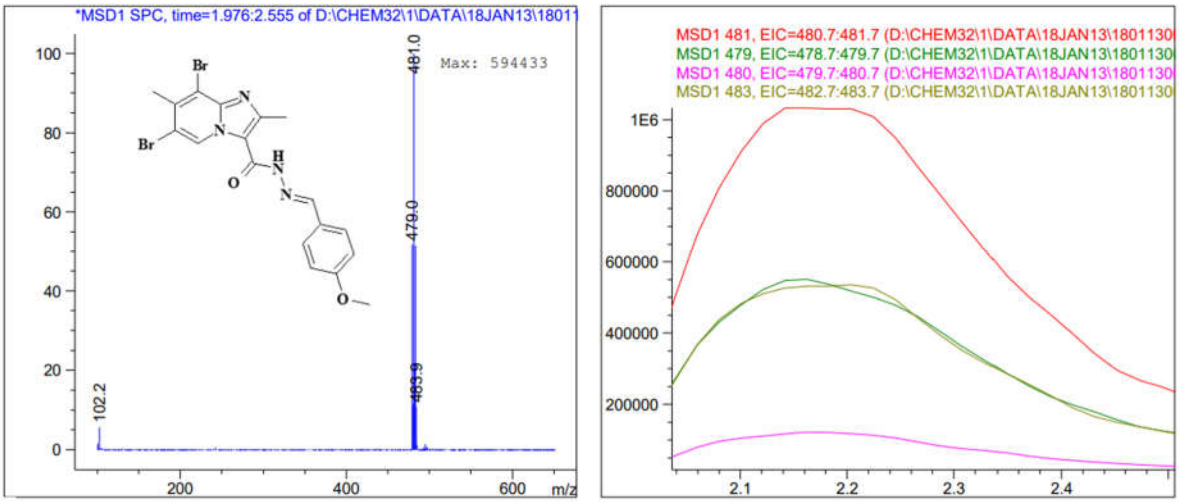

Figure S54. Mass spectra of (E)-6,8-dibromo-N'-(4-methoxybenzylidene)-2,7-dimethylimidazo[1,2-a]pyridine-3-carbohydrazide (**DA-04**).

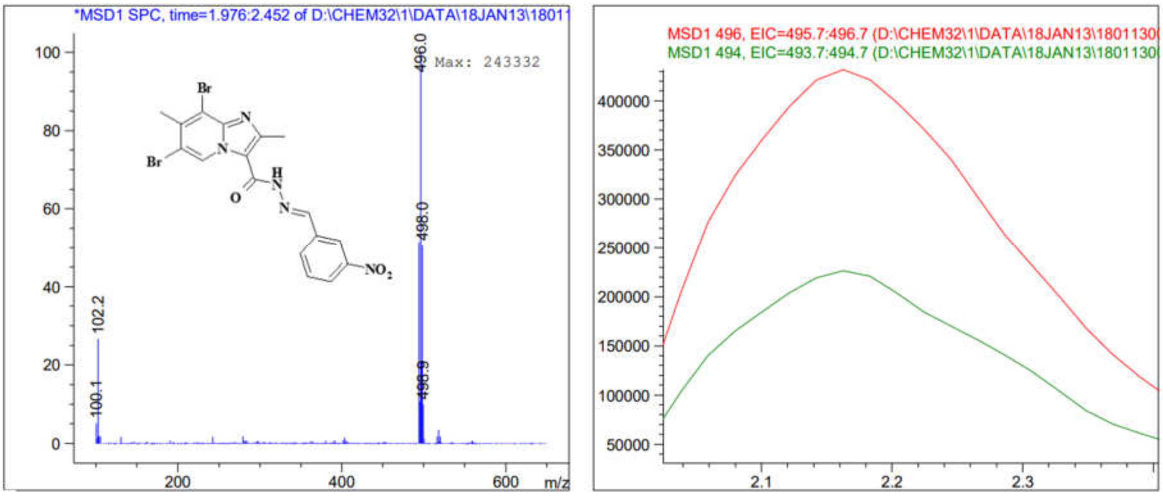

Figure S55. Mass spectra of (E)-6,8-dibromo-2,7-dimethyl-N'-(3-nitrobenzylidene)imidazo[1,2-a]pyridine-3-carbohydrazide (**DA-05**).

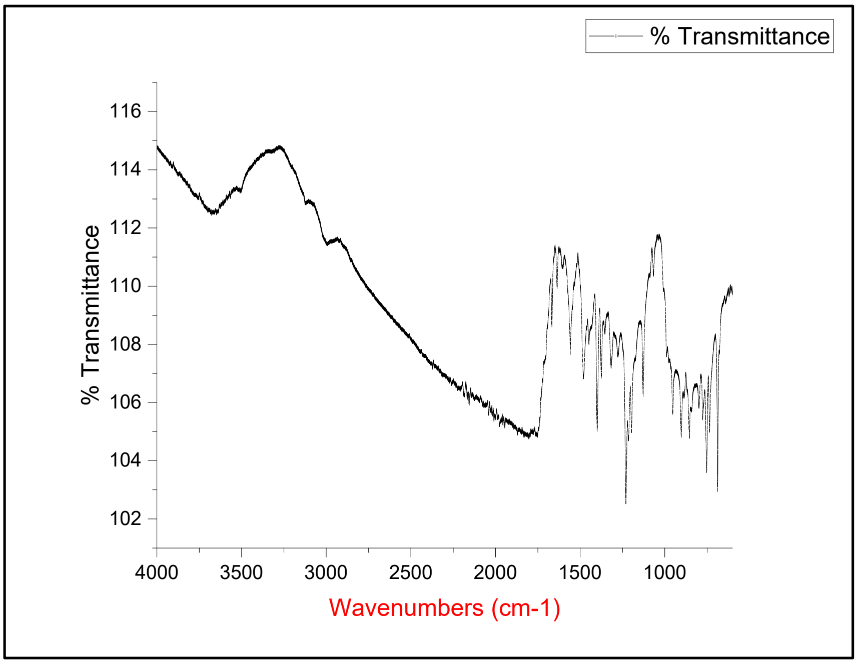

Figure S56. FTIR spectra of (E)-N'-benzylidene-6,8-dibromo-2,7-dimethylimidazo[1,2-a]pyridine-3-carbohydrazide (**DA-01**).

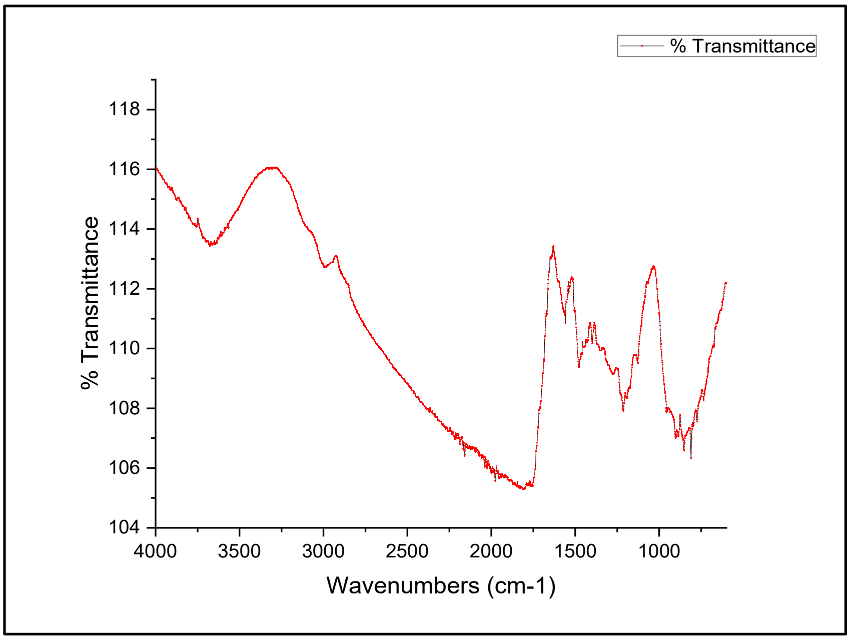

Figure S57. FTIR spectra of (E)-6,8-dibromo-2,7-dimethyl-N'-(4-methylbenzylidene)imidazo[1,2-a]pyridine-3-carbohydrazide (**DA-02**).

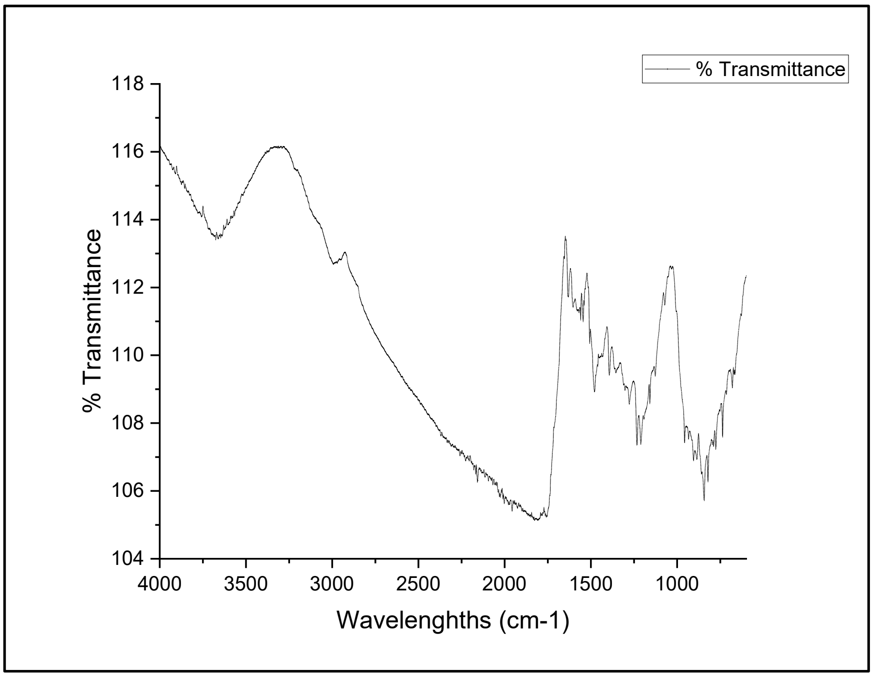

Figure S58. FTIR spectra of (E)-6,8-dibromo-N'-(4-fluorobenzylidene)-2,7-dimethylimidazo[1,2-a]pyridine-3-carbohydrazide (**DA-03**).

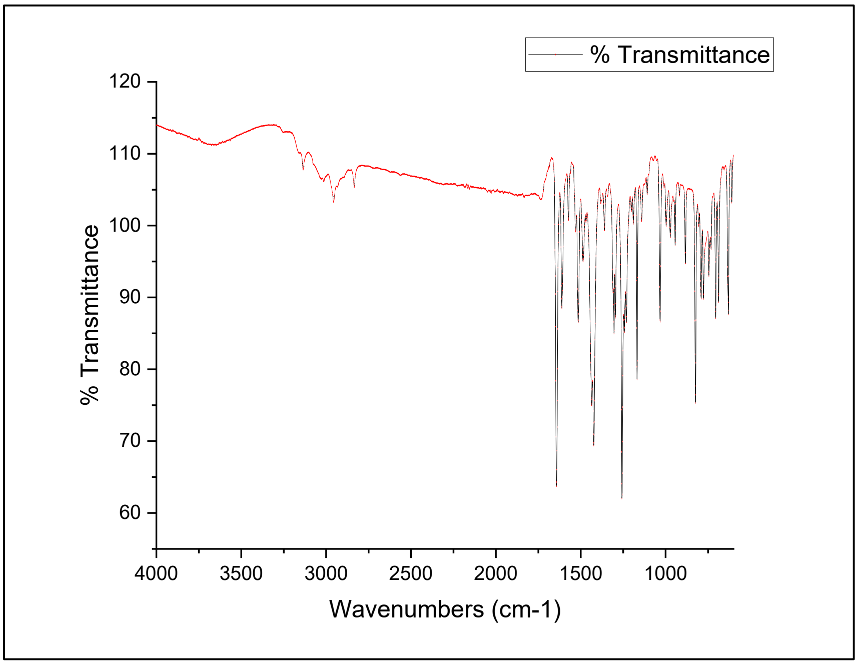

Figure S59. FTIR spectra of (E)-6,8-dibromo-N'-(4-methoxybenzylidene)-2,7-dimethylimidazo[1,2-a]pyridine-3-carbohydrazide (**DA-04**).

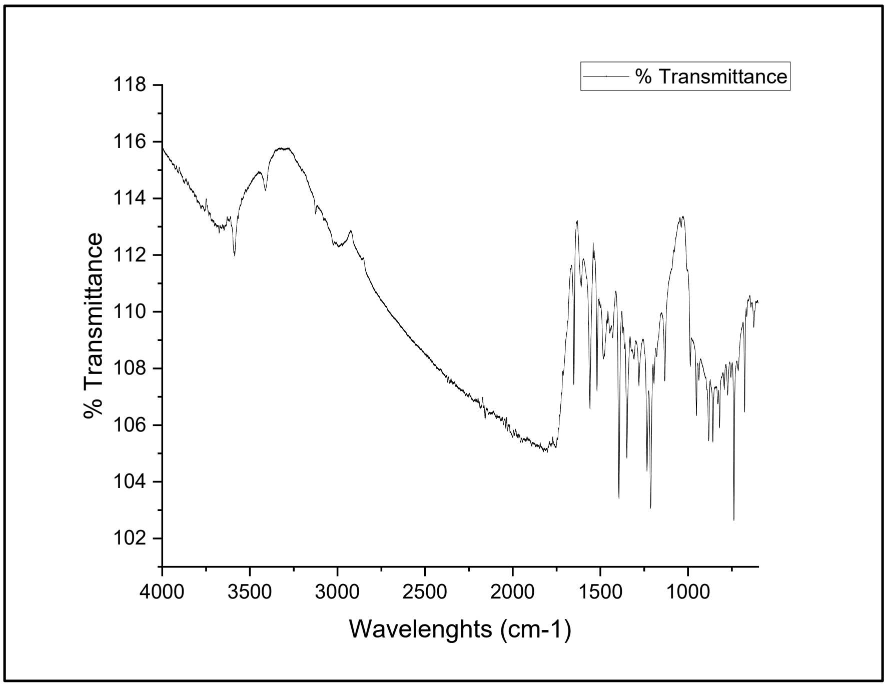

Figure S60. FTIR spectra of (Z)-6,8-dibromo-2,7-dimethyl-N'-(3-nitrobenzylidene)imidazo[1,2-a]pyridine-3-carbohydrazide (**DA-05**).

Biological Assays

The antimicrobial activities of the synthesized compounds were determined against ATCC reference and clinical microbial strains, i.e., *bacterial strain, Klebsiella pneumoniae ATCC 4352 and Bacillus subtilis ATCC 6051,* using as positive control the antibiotic Ciprofloxacin. Microbial suspensions of 1.5×10^8 CFU/mL corresponding to 0.5 McFarland density obtained from 15–18 h bacterial cultures developed on solid media were used in our experiments. The antimicrobial activity was tested on Mueller-Hinton Agar (MHA) medium, while a Yeast Peptone Glucose (YPG) medium was used in case of *C. albicans*. The obtained compounds were solubilized in DMSO and the starting stock solution was of 5000 μg/mL concentration. The qualitative screening was performed by an adapted disk diffusion method as previously reported.

The quantitative assay of the antimicrobial activity was performed by the liquid medium microdilution method, in 96 multi-well plates, in order to establish the minimal inhibitory concentration (MIC). In this purpose, serial two-fold dilutions of the compounds ranging between 5000 and 4.8 μg/mL were performed in a 200 μL volume of broth and each well was seeded with 50 μL of microbial inoculum. Sterility control (wells containing only culture medium) and culture controls (wells containing culture medium seeded with the microbial inoculum) were used. The influence of the DMSO solvent was also quantified in a series of wells containing DMSO, diluted accordingly with the dilution scheme used for the compounds and for the positive controls. The plates were incubated for 24 h at 37 °C, and MIC values were considered as the lowest concentration of the tested compound that inhibited the visible growth of the microbial cultures incubated overnight. The visual determinations have been confirmed by absorbance measurements performed at 600 nm with an ELISA reader Apollo LB 911, the MIC value corresponding to an absorbance value significantly lower than that obtained for the culture control.

Table S1. The results of the qualitative assay of the antimicrobial activity of the tested compounds by using an adapted diffusion assay (the growth inhibition diameters were measured and expressed in mm).

**Comp.ID**	**100 ug/mL (*Klebsiella pneumoniae ATCC 4352)***	**100 ug/mL *Bacillus subtilis ATCC 6051***	**Gaps**
SM-IMP-01	7.9	9.9	**4.55**
SM-IMP-02	11	13.2	**4.43**
SM-IMP-03	9.4	11	4.62
SM-IMP-04	10.7	7	4.46
SM-IMP-05	10	12.2	**4.45**
SM-IMP-06	3.6	6.6	4.58
SM-IMP-07	3	5.8	4.65
SM-IMP-08	10.2	13.3	4.47
SM-IMP-09	9.1	11.7	4.47
SM-IMP-10	8.6	10.8	4.49
SM-IMP-11	7.7	9.5	4.49
SM-IMP-12	3.2	5.6	4.65
SM-IMP-13	2.9	4.7	**4.69**
DA-01	9.6	11.3	4.17
DA-02	10.6	14.9	4.13
DA-03	11.2	13	4.15
DA-04	12.9	15.3	4.01
DA-05	13.7	17.5	**3.24**
Ciprofloxacin	16	21	NA

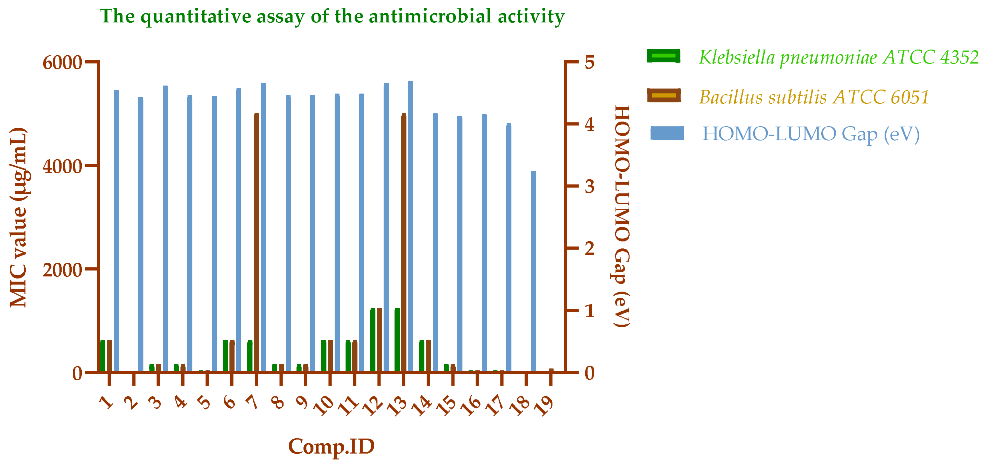

Figure S61. The results of the quantitative assay of the antimicrobial activity of tested compounds 1-18 as (SM-IMP-01) to SM-IMP-13), and (DA-01-05), respectively. Wherein, 19 was used as a standard drug, Ciprofloxacin and the corresponding MIC value (µg/mL).

Table S2. The results of the quantitative assay of the antimicrobial activity of tested compounds 1-18 as (SM-IMP-01) to SM-IMP-13), and (DA-01-05), respectively. Wherein, 19 was used as a standard drug, Ciprofloxacin and the corresponding MIC value (μg/mL).

Comp.ID	Klebsiella pneumoniae ATCC 4352			Bacillus subtilis ATCC 6051			HOMO-LUMO Gap (eV)		
1	625	0	1	625	0	1	4.55	0	1
2	4.8	0	1	4.8	0	1	4.43	0	1
3	156	0	1	156	0	1	4.62	0	1
4	156	0	1	156	0	1	4.46	0	1
5	39	0	1	39	0	1	4.45	0	1
6	625	0	1	625	0	1	4.58	0	1
7	625	0	1	5000	0	1	4.65	0	1
8	156	0	1	156	0	1	4.47	0	1
9	156	0	1	156	0	1	4.47	0	1
10	625	0	1	625	0	1	4.49	0	1
11	625	0	1	625	0	1	4.49	0	1
12	1250	0	1	1250	0	1	4.65	0	1
13	1250	0	1	5000	0	1	4.69	0	1
14	625	0	1	625	0	1	4.17	0	1
15	156	0	1	156	0	1	4.13	0	1
16	39	0	1	39	0	1	4.15	0	1
17	39	0	1	39	0	1	4.01	0	1
18	4.8	0	1	4.8	0	1	3.24	0	1
19	4.8	0	1	78	0	1	0	0	1
